# Prevalence and predictors of vitamin D deficiency in young African children

**DOI:** 10.1186/s12916-021-01985-8

**Published:** 2021-05-20

**Authors:** Reagan M. Mogire, Alireza Morovat, John Muthii Muriuki, Alexander J. Mentzer, Emily L. Webb, Wandia Kimita, Francis M. Ndungu, Alex W. Macharia, Clare L. Cutland, Sodiomon B. Sirima, Amidou Diarra, Alfred B. Tiono, Swaib A. Lule, Shabir A. Madhi, Manjinder S. Sandhu, Andrew M. Prentice, Philip Bejon, John M. Pettifor, Alison M. Elliott, Adebowale Adeyemo, Thomas N. Williams, Sarah H. Atkinson

**Affiliations:** 1 Kenya Medical Research Institute (KEMRI) Centre for Geographic Medicine Coast, KEMRI-Wellcome Trust Research Programme, Kilifi, Kenya; 2 KEMRI-Wellcome Trust Research Programme - Accredited Research Centre, Open University, Kilifi, Kenya; 3 Department of Clinical Biochemistry, Oxford University Hospitals, Oxford, UK; 4 Wellcome Centre for Human Genetics, Nuffield Department of Medicine, University of Oxford, Oxford, UK; 5 Li Ka Shing Centre for Health Information and Discovery, Big Data Institute, University of Oxford, Oxford, UK; 6 Medical Research Council (MRC) Tropical Epidemiology Group, Department of Infectious Disease Epidemiology, London School of Hygiene and Tropical Medicine, London, UK; 7 African Leadership in Vaccinology Expertise (Alive), Faculty of Health Sciences, University of the Witwatersrand, Johannesburg, South Africa; 8 Groupe de Recherche Action en Sante (GRAS), 06, 06 BP 10248, Ouagadougou, Burkina Faso; 9 Medical Research Council/Uganda Virus Research Institute and London School of Hygiene and Tropical Medicine Uganda Research Unit, Entebbe, Uganda; 10 South African Medical Research Council Vaccines and Infectious Diseases Analytical Research Unit, Faculty of Health Sciences, University of the Witwatersrand, Johannesburg, South Africa; 11 Wellcome Sanger Institute, Hinxton, Cambridge, UK; 12 MRC Unit The Gambia at London School of Hygiene and Tropical Medicine, Banjul, The Gambia; 13 Centre for Tropical Medicine and Global Health, Nuffield Department of Medicine, University of Oxford, Oxford, UK; 14 South African Medical Research Council/Wits Developmental Pathways for Health Research Unit, Department of Paediatrics, University of the Witwatersrand, Johannesburg, South Africa; 15 Department of Clinical Research, London School of Hygiene and Tropical Medicine, London, UK; 16 Centre for Research on Genomics and Global Health, National Human Genome Research Institute, National Institutes of Health, South Drive, MSC 5635, Bethesda, Maryland 20891-5635 USA; 17 Department of Infectious Diseases and Institute of Global Health Innovation, Imperial College, London, UK; 18 Department of Paediatrics, University of Oxford, Oxford, UK

**Keywords:** 25-hydroxyvitamin D, Vitamin D deficiency, Africa, Children, Nutrition, Vitamin D binding protein, *GC* genotype

## Abstract

**Background:**

Children living in sub-Saharan Africa have a high burden of rickets and infectious diseases, conditions that are linked to vitamin D deficiency. However, data on the vitamin D status of young African children and its environmental and genetic predictors are limited. We aimed to examine the prevalence and predictors of vitamin D deficiency in young African children.

**Methods:**

We measured 25-hydroxyvitamin D (25(OH)D) and typed the single nucleotide polymorphisms, rs4588 and rs7041, in the *GC* gene encoding the vitamin D binding protein (DBP) in 4509 children aged 0–8 years living in Kenya, Uganda, Burkina Faso, The Gambia and South Africa. We evaluated associations between vitamin D status and country, age, sex, season, anthropometric indices, inflammation, malaria and DBP haplotypes in regression analyses.

**Results:**

Median age was 23.9 months (interquartile range [IQR] 12.3, 35.9). Prevalence of vitamin D deficiency using 25(OH)D cut-offs of < 30 nmol/L and < 50 nmol/L was 0.6% (95% CI 0.4, 0.9) and 7.8% (95% CI 7.0, 8.5), respectively. Overall median 25(OH)D level was 77.6 nmol/L (IQR 63.6, 94.2). 25(OH)D levels were lower in South Africa, in older children, during winter or the long rains, and in those with afebrile malaria, and higher in children with inflammation. 25(OH)D levels did not vary by stunting, wasting or underweight in adjusted regression models. The distribution of Gc variants was Gc1f 83.3%, Gc1s 8.5% and Gc2 8.2% overall and varied by country. Individuals carrying the Gc2 variant had lower median 25(OH)D levels (72.4 nmol/L (IQR 59.4, 86.5) than those carrying the Gc1f (77.3 nmol/L (IQR 63.5, 92.8)) or Gc1s (78.9 nmol/L (IQR 63.8, 95.5)) variants.

**Conclusions:**

Approximately 0.6% and 7.8% of young African children were vitamin D deficient as defined by 25(OH)D levels < 30 nmol/L and < 50 nmol/L, respectively. Latitude, age, season, and prevalence of inflammation and malaria should be considered in strategies to assess and manage vitamin D deficiency in young children living in Africa.

**Supplementary Information:**

The online version contains supplementary material available at 10.1186/s12916-021-01985-8.

## Background

Vitamin D deficiency is estimated to be common worldwide [1], including in Africa [2]. Vitamin D deficiency is an important public health problem due to its link with a growing number of diseases [1]. Children may be at a higher risk of low 25-hydroxyvitamin D (25(OH)D) levels and related diseases [3] including rickets, infectious diseases and impaired growth and development [1, 3]. Young children living in Africa have a high burden of nutritional rickets [4], infectious diseases, and account for more than half of all under-5-year mortality worldwide [5].

Few studies have investigated the prevalence of vitamin D deficiency in young African children and most have followed a case-control design to determine associations with specific disease conditions [2, 4, 6, 7]. Population-based studies include a study from Nigeria with 218 pre-school children aged between 6 and 35 months [8] and two studies from Tanzania, one with 581 infants aged 6 months [9] and another with 948 HIV-exposed (uninfected) infants [10]. Similarly, little is also known about the risk factors for vitamin D deficiency in African children. A single study found that 25(OH)D levels increased with age in 21 infants of Malawian mothers living with HIV [6], and seasonal variation in vitamin D status has been reported in school children in Algeria (*n*=435) and South Africa (*n*=385) [11, 12]. Vitamin D deficiency was associated with severe wasting in 21 young Kenyan children with rickets [4], but was not associated with sex, stunting, underweight or wasting in 581 Tanzanian infants [9]. Studies have reported conflicting findings regarding associations between vitamin D status and inflammation [13, 14]; however, only a single study has been conducted in African children [7]. Similarly, only a few studies have evaluated the relationship between vitamin D status and malaria, with mixed findings [9, 10, 15].

Genetic polymorphisms in the group-specific component gene, *GC*, in the 4^th^ chromosome that codes for Gc globulin (Gc), also known as the vitamin D binding protein (DBP), have been associated with vitamin D status and many pathophysiological conditions [16]. More than 85% of circulating vitamin D metabolites (including 25(OH)D) are bound to DBP [16]. The combination of two *GC* SNPs (rs7041 and rs4588) give rise to three major DBP variants with different amino acid and glycosylation characteristics, Gc1f, Gc1s and Gc2, and six DBP haplotypes: Gc1f/1f, Gc1f/1 s, Gc1f/2, Gc1s/1 s, Gc1s/2 and Gc2/2 [16]. The DBP variants have been reported to differ in binding affinity and concentrations [16–18]. The Gc1f allele is most frequent in individuals of African ancestry, while Gc1s is more common in Europeans [17]. Nevertheless, little is known about the genetics of vitamin D and how it is related to vitamin D status in populations living in Africa. To our knowledge, only two small genetic studies in The Gambia (*n* = 237 and *n* = 18) have assessed the association between 25(OH)D levels and DBP haplotypes in Africa [19, 20].

Information on the prevalence and predictors of low vitamin D status in young African children is important in guiding public health policy, however, this information is limited in African populations. In the current study, we measured 25-hydroxyvitamin D (25(OH)D) levels in 4509 children living in Kenya, Uganda, Burkina Faso, The Gambia and South Africa and evaluated the prevalence and predictors of vitamin D deficiency.

## Methods

### Study cohorts

This study included young children living in Kenya (*n* = 1361), Uganda (*n* = 1301), Burkina Faso (*n* = 329), The Gambia (*n* = 629) and South Africa (*n* = 889). Details of these cohorts have previously been described [21–25] and are briefly summarised below.

#### Kilifi, Kenya (3.5° S, 39.9° E)

This is an ongoing community-based cohort aimed at evaluating immunity to malaria in children [21]. Children were followed up from birth to eight years with weekly follow-ups and annual cross-sectional surveys during which anthropometric measurements and blood samples were collected. Levels of 25(OH)D, CRP and malaria parasitemia were measured in plasma samples from a single cross-sectional survey, based on the availability of samples archived at − 80 °C.

#### Entebbe, Uganda (0.1° N, 32.5° E)

The Entebbe Mother and Baby Study (EMaBS) is a prospective birth cohort study that was originally designed as a randomised controlled trial (ISRCTN32849447) aimed at evaluating the effects of helminths and anthelmintic treatment during pregnancy and early childhood on immunological responses to routine vaccinations and incidence of infections in childhood [22]. Anthropometry and blood samples were collected at birth, and at subsequent annual visits. Laboratory assays were conducted in samples from a single annual visit based on the availability of stored samples archived at – 80 °C.

#### Banfora, Burkina Faso (10.6° N, 4.8° W)

The VAC050 ME-TRAP Malaria Vaccine trial tested the effectiveness, safety and immunogenicity of a malaria vaccine in children between the ages of six and 17 months [23]. Anthropometry and blood samples were collected at multiple time-points after receipt of the experimental vaccine. Levels of 25(OH)D, CRP and malaria parasitaemia were measured on stored plasma samples archived at – 80 °C.

#### West Kiang, The Gambia (13.3° N, 16.0° W)

This study included children aged between two and six years recruited from 10 rural villages in the West Kiang region of The Gambia as previously described [25]. Anthropometry and biomarkers were measured in samples from a single cross-sectional survey at the start of the malaria season.

#### Soweto, South Africa (26.2° S, 27.9° E)

The Soweto Vaccine Response Study included infants of African heritage recruited from vaccine trials [24]. This study used stored plasma samples collected from infants that had received all of their routine Expanded Program on Immunization vaccines. The study was conducted in a non-malaria-endemic region and anthropometry and haemoglobin levels were not measured in this cohort.

### Laboratory assays

Assays of 25(OH)D (chemiluminescent microparticle immunoassay, Abbot Architect) and C-reactive protein (CRP) (MULTIGENT CRP Vario assay, Abbot Architect) and α1-antichymotrypsin (ACT) (immunoturbidimetry, Cobas Mira Plus Bioanalyser, Roche) were performed. A verification of the 25(OH)D assay and the comparison of its results with those from an LC/MS method have been published previously [26]. In-house assessments of the assay showed heparinized plasma to give results that were on average 5.1% lower than those obtained on matching serum. The assay’s performance was monitored by 12-hourly quality control checks, with overall CVs that ranged from 2.8% to 7.9% for mean 25(OH)D concentrations ranging from 21 to 116 nmol/L (Additional file 9: Figure S1). Over a 6-month period that spanned the 20-week period of analyses, three sets of external quality assurance (DEQAS) data showed the method to have a mean (SD) bias of − 2.7% (7.6) against the all-laboratory trended values, and one of − 0.4% (7.7) against the target values. Malaria parasitaemia was detected using Giemsa-stained thick and thin blood smears.

### Definitions

Vitamin D status was defined using 25(OH)D cut-offs of < 30 nmol/L, < 50 nmol/L, and 50–75 nmol/L, as adapted from the Endocrine Society and the US Institute of Medicine guidelines [27–29]. Inflammation was defined as CRP level > 5 mg/L or ACT > 0.6 g/L [30]. Malaria parasitaemia was defined as the presence of asexual malaria parasites at any density. Height-for-age *z*-scores (HAZ), weight-for-age *z*-scores (WAZ), and weight-for-height z-scores (WHZ) were computed using the 2006 WHO child growth standards [31]. Stunting was defined as HAZ < − 2, underweight as WAZ < − 2 and wasting as WHZ < − 2. Season was defined using 3 monthly intervals (1st season, December, January, February; 2nd, March, April, May; 3rd, June, July, August; 4th, September, October, November). In South Africa, the seasons correspond to summer, autumn, winter and spring; in Uganda and Kenya, there are two rainy and two dry seasons; and in Burkina Faso and The Gambia, there is a single rainy and a single dry season, although the timing of the rains is often unpredictable and may vary [12, 32, 33].

### Genotyping and SNP quality control

Genomic DNA from study participants were genotyped using genome-wide SNP arrays (see Additional file 1: Supplementary Methods for more details). Two *GC* SNPs (rs7041 and rs4588) were retrieved from imputed data and their combinations used to classify participants into Gc variants; Gc1f (T and C), Gc1s (G and C) and Gc2 (T and A) and six DBP haplotypes; Gc1f/f (TT, CC), Gc1f/s (TG, CC), Gc1f/2 (TT, CA), Gc1s/s (GG, CC), Gc1s/2 (TG, CA), and Gc2/2 (TT, AA).

### Statistical analyses

All statistical analyses were conducted using Stata Statistical Software: Release 15 (College Station, TX: StataCorp LLC) and R version 3.5.1 (https://www.R-project.org/). 25(OH)D levels were natural log (ln)-transformed to normalise their distributions in regression analyses. Medians and geometric means for 25(OH)D levels were used to summarise average 25(OH)D levels for different groups. Between-group differences in median 25(OH)D levels were tested using Wilcoxon rank-sum test (two categories) and Kruskal-Wallis equality-of-populations rank test (more than two categories). Linear and logistic regression analyses were performed to evaluate the association between vitamin D status (ln-25(OH)D levels and 25(OH)D levels of < 50 and between 50 and 75 nmol/L compared to > 75 nmol/L) and country, age, sex, season, stunting, underweight, wasting, inflammation, malaria, and DBP haplotypes and variants. Since few children had 25(OH)D levels < 30 nmol/L, further analyses did not include this group. Multivariable regression analyses were adjusted for age, sex, season, inflammation, and study site, as appropriate.

We further searched PubMed and Embase for published studies that measured serum 25(OH)D levels in healthy children aged 0–8 years in Africa without date of publication or language restrictions. The search strategy is presented in Additional file 2: Table S1. We then carried out meta-analyses of low vitamin D status categories (25(OH)D levels < 50 and < 75 nmol/L) and mean 25(OH)D levels using random effects models (‘*meta*’ R package).

### Role of the funding source

The funders had no role in the study design, data collection, data analysis, data interpretation, or writing of the report. The corresponding authors had full access to all the data and had final responsibility for the decision to submit for publication.

## Results

### Characteristics of study participants

A total of 4509 infants and children with an age range of 0.2 months to 8 years and a median age of 23.9 months (interquartile range 12.3, 35.9) were included in the study (Table 1). Approximately half (49.1%) of children were female. Overall prevalence of stunting, underweight and wasting was 25.4%, 15.6% and 6.4%, respectively, and varied by country with the highest prevalence observed in Kenyan children. Overall prevalence of inflammation and asymptomatic malaria was 22.8% and 13.5%, respectively, and varied by country with the highest prevalence observed in Burkina Faso (33.9% and 21.1%, respectively) (Table 1).

**Table 1:** Characteristics of study participants

	Overall	Kenya	Uganda	Burkina Faso	The Gambia	South Africa
**No. of participants (%)**	4509 (100%)	1361 (30.1%)	1301 (28.9%)	329 (7.3%)	629 (13.9%)	889 (19.7%)
**Median 25(OH)D nmol/L (IQR)**^a^	77.6 (63.6, 94.2)	81.0 (66.3, 101.6)	78.6 (65.1, 94.5)	78.4 (64.5, 91.3)	71.2 (59.1, 84.2)	76.2 (60.6, 91.9)
**Vitamin D status**						
25(OH)D > 150 nmol/l	79/4509 (1.8%)	51/1361 (3.7%)	17/1301 (0.1%)	4/329 (1.3%)	1/629 (0.2%)	6/889 (0.7%)
25(OH)D > 75 nmol/l	2485/4509 (55.1%)	815/1361 (59.9%)	756/1301 (58.1%)	186/329 (56.5%)	265/629 (42.1%)	463/889 (52.1%)
25(OH)D 50–75 nmol/l	1674/4509 (37.1%)	464/1361 (34.1%)	479/1301 (36.8%)	123/329 (37.4%)	302/629 (48.0%)	306/889 (34.4%)
25(OH)D < 50 nmol/l	350/4509 (7.8%)	82/1361 (6.0%)	66/1301 (5.1%)	20/329 (6.1%)	62/629 (9.9%)	120/889 (13.5%)
25(OH)D < 30 nmol/l	28/4509 (0.6%)	4/1361 (0.3%)	5/1301 (0.4%)	0/329 (0%)	2/629 (0.3%)	17/889 (1.9%)
**Median age (months)**	23.9 (12.3, 35.9)	19.8 (12.7, 36.8)	24.1 (23.9, 35.9)	23.4 (19.7, 26.4)	46.6 (35.2, 58.7)	12.0 (11.9, 12.1)
**Age categories (months)**						
< 12	816/4509 (18.1%)	300/1361 (22.0%)	24/1301 (1.8%)	19/329 (5.8%)	-	473/889 (53.2%)
12–24	1597/4509 (35.4%)	555/1361 (40.8%)	440/1301 (33.8%)	172/329 (52.3%)	15/629 (2.4%)	415/889 (46.7%)
24–36	1029/450 (22.8%)	153/1361 (11.2%)	587/1301 (45.1%)	138/329 (42.0%)	150/629 (23.9%)	1/889 (0.1%)
36–48	478/4509 (10.6%)	146/1361 (10.7%)	167/1301 (11.8%)	-	165/629 (26.2%)	-
48+	589/4509 (13.1%)	207/1361 (15.2%)	83/1301 (6.4%)	-	299/629 (47.5%)	-
**Sex:** females	2216/4509 (49.1%)	671/1361 (49.3%)	641/1301 (49.3%)	161/329 (48.9%)	297/629 (47.2%)	446/889 (50.2%)
**Season**^eb^						
Summer/short rains/dry	867/4503 (19.3%)	285/1361 (20.9%)	331/1296 (25.5%)	72/329 (21.9%)	-	179/889 (18.1%)
Autumn/dry	1475/4503 (32.8%)	896/1361 (65.8%)	295/1296 (22.8%)	123/329 (37.4%)	-	161/889 (18.1%)
Winter/long rains	1361/4503 (30.2%)	86/1361 (6.3%)	330/1296 (25.5%)	129/329 (39.2%)	536/628 (85.4%)	280/889 (31.5%)
Spring/dry	800/4503 (17.8%)	94/1361 (6.9%)	340/1296 (26.2%)	5/329 (1.5%)	92/628 (14.7%)	269/889 (30.3%)
**Nutritional status**^c^						
Stunted	581/2289 (25.4%)	99/208 (47.6%)	203/1282 (15.8%)	103/307 (33.5%)	176/492 (35.8%)	n/a
Underweight	389/2487 (15.6%)	102/389 (26.2%)	103/1296 (8.0%)	58/309 (18.8%)	126/493 (25.6%)	n/a
Wasted	147/2285 (6.4%)	24/205 (11.7%)	59/1281 (4.6%)	20/307 (6.5%)	44/492 (8.9%)	n/a
**Inflammation**^d^	1019/4469 (22.8%)	363/1344 (27.0%)	306/1285 (23.8%)	109/322 (33.9%)	85/629 (13.5%)	156/889 (17.6%)
**Malaria**^e^	445/3293 (13.5%)	227/1082 (20.8%)	89/1280 (7.0%)	64/303 (21.1%)	65/628 (10.4%)	n/a

South African children were not exposed to malaria

*IQR* inter-quartile range, *n/a* not available, *25(OH)D* 25-hydroxyvitamin D

^a^Medians (interquartile ranges) are presented. ^b^Seasons were based on 3 monthly intervals: 1st season, December to February; 2nd season, March to May; 3rd season, June to August; 4th season, September to November. In South Africa, the seasons correspond to summer, autumn, winter and spring, respectively, in Uganda and Kenya there are two rainy and two dry seasons and in Burkina Faso and The Gambia there is a single rainy and dry season. However, timing of the rains is often unpredictable and may vary from these times. ^c^Stunted was defined as height-for-age *Z* score < − 2; underweight as weight-for-age *Z* score < − 2, wasted as weight-for-height *Z* score < − 2 (denominator number varied because anthropometry data was not available for South African children). ^d^Inflammation as CRP > 5 mg/L or ACT > 0.6 g/L. ACT, but not CRP, was available for The Gambia. ^e^Malaria as the presence of *P. falciparum* parasites on blood film

### Vitamin D status

**Fig. 1:**
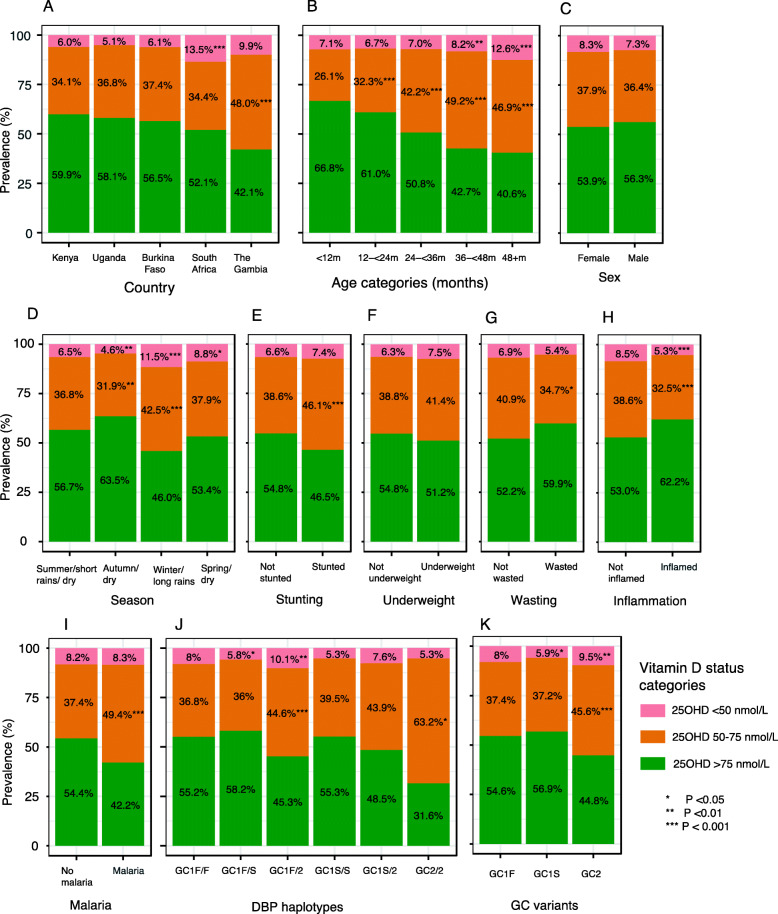
Prevalence of vitamin D categories by country (**a**), age categories (**b**), sex (**c**), season (**d**), stunting (**e**), underweight (**f**), wasting (**g**), inflammation (**h**), malaria status (**i**), and vitamin D binding protein (DBP) haplotypes (**j**) and variants (**k**). Season was based on 3 monthly intervals. In South Africa the seasons are summer, autumn, winter and spring, in Uganda and Kenya there are two rainy seasons and in Burkina Faso and The Gambia a single rainy season. Stunting was defined as height-for-age *Z* score < − 2; underweight as weight-for-age *Z* score < − 2, wasting as weight-for-height *Z* score < − 2; inflammation as CRP > 5 mg/L or ACT > 0.6 g/L (ACT, but not CRP was available for The Gambia); malaria as presence of *P. falciparum* parasites on blood film. *Prtest* (STATA) was used to test the significance in the difference in the proportion of low vitamin status (25(OH)D levels < 50 or 50–75 nmol/L) within each category with the first category as the reference

Overall median 25(OH)D level was 77.6 nmol/L (IQR 63.6, 94.2), and geometric mean 25(OH)D level was 77.0 nmol/L (95% CI 76.3, 77.7). Prevalence of vitamin D deficiency defined by 25(OH)D levels of < 50 nmol/L or < 30 nmol/L were 7.8% (350/4509) and 0.6% (28/4509), respectively (Table 1). A total of 1674 children (37.1%) had 25(OH)D levels between 50 and 75 nmol/L. The prevalence of vitamin D deficiency varied by country, with the highest prevalence observed in South African children (Table 1 and Fig. 1). About 1.8% (79/4509) of children had 25(OH)D levels above 150 nmol/L (51 Kenyan, 17 Ugandan, four Burkinabe, one Gambian and six South African children).

### Vitamin D status is associated with age and season, but not with nutritional status

25(OH)D levels decreased with increasing age across all age groups (Additional file 3: Table S2 and Additional file 10: Figure S2), even after adjustment for potential confounders in multivariable regression analyses (Table 2). Approximately 4.5% of the observed variation (*R*^2^ = 0.045) in 25(OH)D levels was explained by age (Table 2). In a multivariable linear regression analysis, study site, age, sex, season and inflammation accounted for 12% (*R*^2^ = 0.12) of observed variation in 25(OH)D levels. Each additional year of age increased the odds of 25(OH)D levels < 50 and 50–75 nmol/L by 69% (OR 1.69, [95% CI 1.52, 1.89]) and 43% (OR 1.43, [95% CI 1.34, 1.52]), respectively (Additional file 4: Table S3). Vitamin D deficiency (25(OH)D < 50 nmol/L) was more prevalent during the South African winter and the long rains in sub-Saharan Africa (Fig. 1 and Additional file 4: Table S3). Seasonality explained 3.8% of the variation in 25(OH)D levels (*R*^2^ = 0.038) (Table 2).

**Table 2:** Environmental factors are associated with vitamin D status

	25(OH)D levels (nmol/L)^a^		Unadjusted regression analyses of 25(OH)D levels			Adjusted regression analyses of 25(OH)D levels		Adjusted logistic regression analyses 25(OH)D < 50 nmol/L	
	Medians (IQR)	Means (95% CI)	Beta (95% CI)	***P***	***R***^**2**^	Beta (95% CI)	***P***	OR (95% CI)	***P***
**Age in years**	-	-	− 0.05 (− 0.06, − 0.04)	< 0.0001	0.045	− 0.07 (− 0.08, − 0.07)	< 0.0001	1.69 (1.52, 1.89)	< 0.0001
**Sex**									
Males	77.7 (64.1, 94.5)	77.50 (76.52, 78.49)	Ref.	–		Ref.	–	Ref.	–
Females	77.2 (63.0, 94.0)	76.45 (75.43, 77.48)	− 0.01 (− 0.03, 0.01)	0.15	0.0005	− 0.02 (− 0.03, 0.001)	0.066	1.32 (1.04, 1.68)	0.021
**Country**									
Kenya	81.0 (66.3, 101.6)	81.85 (80.42, 83.30)	Ref.	–		Ref.	–	Ref.	–
Uganda	78.6 (65.1, 94.5)	78.27 (77.02, 79.54)	− 0.04(− 0.07, − 0.02)	0.0002	0.029	0.01 (− 0.02, 0.03)	0.51	0.50 (0.34, 0.74)	0.001
Burkina Faso	78.4 (64.5, 91.3)	77.26 (74.98, 79.61)	− 0.06 (− 0.10, − 0.02)	0.003		− 0.05 (− 0.08, − 0.01)	0.012	0.81 (0.45, 1.44)	0.47
The Gambia	71.2 (59.1, 84.2)	72.78 (71.15, 74.45)	− 0.15 (− 0.18, − 0.12)	< 0.0001		0.09 (0.05, 0.13)	< 0.0001	0.29 (0.17, 0.47)	< 0.0001
South Africa	76.2 (60.6, 91.9)	70.39 (68.95, 71.86)	− 0.12 (− 0.14, − 0.09)	< 0.0001		− 0.15 (− 0.18, − 0.12)	< 0.0001	2.89 (1.94, 4.29)	< 0.0001
**Season**^**b**^									
Summer/short rains/dry	78.8 (64.8, 95.8)	78.77 (77.19, 80.39)	Ref.	–		Ref.	–	Ref.	–
Autumn/dry	83.6 (67.1, 101.1)	82.72 (81.43, 84.02)	0.05 (0.02, 0.07)	0.0002	0.038	0.03 (0.01, 0.06)	0.014	0.65 (0.40, 0.96)	0.032
Winter/long rains	73.3 (60.1, 87.0)	71.14 (69.96, 72.34)	− 0.10 (− 0.13, − 0.08)	< 0.0001		− 0.10 (− 0.12, − 0.07)	< 0.0001	2.55 (1.75, 3.72)	< 0.0001
Spring/dry	76.3 (63.1, 91.4)	75.18 (73.55, 76.84)	− 0.05 (− 0.08, − 0.02)	0.002		− 0.05 (− 0.08, − 0.02)	0.002	1.60 (1.07, 2.38	0.022
**Stunting**^**c**^									
Not stunted	77.1 (63.7, 91.0)	76.13 (75.08, 77.19)	Ref.	–		Ref.	–	Ref.	–
Stunted	73.1 (61.2, 88.4)	73.58 (71.89, 75.31)	− 0.03 (− 0.06, − 0.01)	0.015	0.003	− 0.01 (− 0.04, 0.01)	0.34	1.05 (0.69, 1.58)	0.82
**Underweight**^**d**^									
Not underweight	77.2 (63.8, 91.7)	76.67 (75.72, 77.64)	Ref.	–		Ref.	–	Ref.	–
Underweight	75.6 (61.9, 91.3)	74.90 (72.68, 77.18)	− 0.02 (− 0.06, 0.01)	0.15	0.0008	− 0.004 (− 0.04, 0.03)	0.81	0.88 (0.54, 1.40)	0.58
**Wasting**^**e**^>									
Not wasted	76.0 (62.9, 90.6)	75.32 (74.40, 76.26)	Ref.	–		Ref.	–	Ref.	–
Wasted	79.2 (63.6, 94.5)	77.81 (74.45, 81.32)	0.03 (− 0.02, 0.08)	0.19	0.0008	0.04 (− 0.01, 0.08)	0.12	0.64 (0.29, 1.38)	0.25
**Inflammation**^f^									
Without inflammation	76.4 (62.7, 92.3)	75.53 (74.74, 76.3)	Ref.	–		Ref.	–	Ref.	–
With inflammation	81.9 (68.0, 99.5)	82.11 (80.55, 83.69)	0.08 (0.06, 0.11)	< 0.0001	0.012	0.07 (0.05, 0.09)	< 0.0001	0.59 (0.43, 0.81)	0.001
**Malaria**^g^									
Without malaria	77.1 (63.1, 92.4)	76.01 (75.26, 76.77)	Ref.	–		Ref.	–	Ref.	–
With malaria	71.3 (58.9, 85.4)	71.63 (69.68, 73.63)	− 0.06 (− 0.09, − 0.03)	0.0001	0.004	− 0.04 (− 0.07, − 0.01)	0.02	1.02 (0.65, 1.61)	0.93

Correlation coefficients and p values were obtained from linear regression analyses and odds ratios from logistic regression analyses. 25(OH)D levels were ln-transformed in linear regression analyses to make them normally distributed. Covariates in the multivariable linear regression models included age, sex, season, inflammation and study site, as appropriate

*25(OH)D* 25-hydroxyvitamin D, *n/a* not available, *OR* odds ratio, *95% CI* 95% confidence interval

^a^Medians (IQR) and geometric means are presented.^b^Season was based on 3 monthly intervals. In South Africa, the seasons were summer, autumn, winter and spring; in Uganda and Kenya, there are two rainy seasons; and in Burkina Faso and The Gambia, there is a single rainy season. ^c^Stunting was defined as height-for-age *Z* score < − 2; ^d^underweight as weight-for-age *Z* score < − 2; ^e^wasting as weight-for-height *Z* score < − 2; ^f^inflammation as CRP > 5 mg/L or ACT > 0.6 g/L and ^g^malaria as presence of *P. falciparum* parasites on blood film. Analyses by country are presented in Additional file 4: Table S3 (logistic regression) Additional file 5: Table S4 (unadjusted linear regression), and Additional file 6: Table S5 (adjusted linear regression)

Median 25(OH)D levels were lower in stunted children in univariable analyses, but this association was not observed after adjustment for potential confounding factors in multivariable regression models (Table 2). Overall 25(OH)D levels were not associated with sex, underweight or wasting although girls had a 32% higher risk of 25(OH)D levels of <50 nmol/L (Table 2 and Fig. 1), a finding that was mainly observed in The Gambia. Findings are presented by individual countries in Additional file 3: Tables S2, Additional file 4: Table S3, Additional file 5: Table S4 and Additional file 6: Table S5.

### 25(OH)D levels are higher with inflammation and lower with malaria

Children with inflammation (CRP > 5 mg/L or ACT > 0.6 g/L) had higher median 25(OH)D levels (81.9 nmol/L [IQR 68.0, 99.5]) than those without inflammation (76.4 nmol/L [IQR 62.7, 92.3])) (Table 2 and Additional file 10: Figure S2), a difference that was observed in all countries except Burkina Faso (Additional file 3: Table S2). Inflammation explained 1.2% (*R*^2^ = 0.012) of the total variation in 25(OH)D levels (Table 2). Children with inflammation were 42% and 26% less likely to have 25(OH)D levels of < 50 and 50–75 nmol/L, respectively, compared to those with 25(OH)D levels > 75 nmol/L (Additional file 4: Table S3). CRP levels also varied by country (Additional file 11: Figure S3).

Children with asymptomatic malaria parasitaemia had lower median 25(OH)D levels (71.3 nmol/L [IQR 58.9, 85.4]) than those without (77.1 nmol/L (IQR 63.1, 92.4) (Table 2 and Additional file 10: Figure S2). Malaria was further associated with lower vitamin D status in multivariable regression analyses adjusted for potential confounders, although this association was observed only in Kenya (Table 2, Additional file 4: Table S3 and Additional file 6: Table S5). Malaria parasitaemia explained 0.4% of variation in 25(OH)D levels (R^2^ = 0.004) (Table 2).

### Vitamin D binding protein variants are associated with vitamin D status

Overall, the most frequent DBP haplotype was Gc1f/f (69.8%), followed by Gc1f/2 (13.6%), Gc1f/s (13.4%), Gc1s/2 (1.7%), Gc1s/s (1.0%), and least frequent was Gc2/2 (0.6%) (Table 3). The most frequent Gc variant was Gc1f (83.3%), followed by Gc1s (8.5%), and the least frequent was Gc2 (8.2%). Frequencies of DBP haplotypes and variants varied by country, with the highest frequencies of Gc1f, Gc1s and Gc2 observed in South Africa (87.3%), The Gambia (13.1%) and Uganda (10.2%), respectively (Additional file 7: Table S6). Median 25(OH)D levels were lowest in children carrying the Gc2 variant (72.4 nmol/L [IQR 59.4, 86.5]), but did not differ between the Gc1f (77.3 nmol/L [IQR 63.5, 92.8]) and Gc1s variants (78.9 nmol/L [63.8, 95.5]). Median 25(OH)D levels similarly differed by DBP haplotype (Table 3). DBP haplotypes and variants explained 0.9% and 0.4% of the variation in 25(OH)D levels, respectively (Table 3). The Gc2 variant was associated with lower 25(OH)D levels (*β* = − 0.08 [95% CI − 0.11, − 0.06] and a 69% (OR 1.69 [1.23, 2.31]) increased risk of vitamin D deficiency (25(OH)D levels < 50 nmol/L) in adjusted regression analyses (Table 3). The Gc2 variant was similarly associated with the highest prevalence of vitamin D deficiency (Fig. 1). Country-specific analyses are presented in Additional file 7: Table S6).

**Table 3:** Vitamin D binding protein haplotypes and variants are associated with vitamin D status

Combination of genotypes		DBP Haplotype^a^	***n***/total (%)	25(OH)D levels (nmol/L)^c^		Unadjusted regression analyses of 25(OH)D levels			Adjusted regression analyses of 25(OH)D levels		Adjusted regression analyses 25(OH)D < 50 nmol/L	
rs7041	rs4588			Medians (IQR)	Means (95% CI)	Beta (95% CI)	***P***	***R***^**2**^	Beta (95% CI)	***P***	OR (95% CI)	***P***
TT	CC	**Gc1f/f**	2689/3851 (69.8%)	77.7 (63.6, 93.0)	76.7 (75.8, 77.6)	Ref	–	0.009	Ref	–	Ref	–
TG	CC	**Gc1f/s**	514/3851 (13.4%)	79.2 (65.5, 96.0)	78.7 (76.6, 80.8)	0.03 (0.004, 0.05)	0.092		0.03 (− 0.0023, 0.05)	0.077	0.71 (0.47, 1.08)	0.11
TT	CA	**Gc1f/2**	525/3851 (13.6%)	72.7 (60.0, 86.8)	71.6 (69.7, 73.6)	− 0.07 (− 0.10, − 0.04)	< 0.0001		− 0.08 (− 0.11, − 0.05)	< 0.0001	1.79 (1.26, 2.55)	0.001
GG	CC	**Gc1s/s**	38/3851 (1.0%)	81.8 (61.0, 95.7)	77.8 (69.7, 86.9)	0.01 (− 0.08, 0.11)	0.78		0.01 (− 0.09, 0.10)	0.85	0.73 (0.16, 3.29)	0.69
TG	CA	**Gc1s/2**	66/3851 (1.7%)	74.6 (59.4, 87.6)	71.7 (66.1, 77.8)	− 0.07 (− 0.14, − 0.01)	0.081		− 0.08 (− 0.15, − 0.01)	0.026	1.18 (0.43, 3.30)	0.75
TT	AA	**Gc2/2**	19/3851 (0.5%)	63.9 (52.4, 79.7)	65.6 (58.0, 74.1)	− 0.16 (− 0.29, − 0.02)	0.027		− 0.18 (− 0.37, − 0.04)	0.009	2.34 (0.27, 20.40)	0.44
		**Gc variant**^**b**^										
T	C	**Gc1f**	6417/7702 (83.3%)	77.3 (63.5, 92.8)	76.5 (75.9, 77.0)	Ref	–	0.004	Ref	–	Ref	–
G	C	**Gc1s**	656/7702 (8.5%)	78.9 (63.8, 95.5)	77.9 (76.0, 79.7)	0.02 (− 0.01, 0.043)	0.15		0.02 (− 0.01, 0.04)	0.16	0.73 (0.52, 1.06)	0.096
T	A	**Gc2**	629/7702 (8.2%)	72.4 (59.4, 86.5)	71.3 (69.5, 73.1)	− 0.07 (− 0.10, − 0.04)	< 0.0001		− 0.08 (− 0.11, − 0.06)	< 0.0001	1.69 (1.23, 2.30)	0.001

*DBP* vitamin D binding protein, *Gc* group-specific component, *IQR* inter-quartile range, *n/a* not available, *25(OH)D* 25-hydroxyvitamin D, *OR* odds ratio, *95% CI* 95% confidence interval

^a,b^DBP haplotypes and Gc variants are based on combination of rs7041 and rs4588 genotypes. ^c^Median (IQR) and geometric means (95% CI) are presented. Percentage is based on the successfully typed DBP, some participants’ genotype data was not available or failed QC. DBP haplotype and Gc variant frequencies are presented by country in Additional file 7: Table S6

### Meta-analysis

**Fig. 2:**
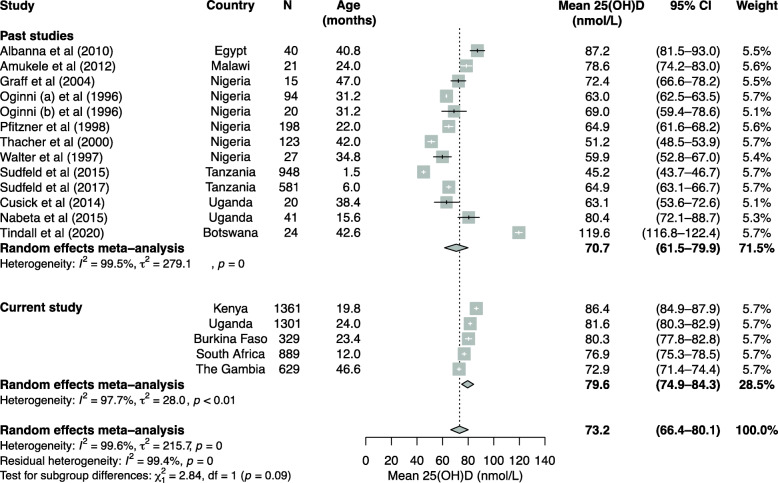
Meta-analysis of studies that evaluated 25(OH)D levels in healthy young children in Africa. For case-control studies, we only included mean 25(OH)D levels of healthy controls in the meta-analysis. Means of age in months are presented. Studies that only measured 25(OH)D levels in cord blood, reported only median values, or did not report estimates from young children (aged 0–8 years) separately from older children were excluded from these analyses. Details of the studies are presented in Additional file 8: Table S7

Out of 18 previous studies that assessed the vitamin D status of African children aged 0–8 years (Additional file 8: Table S7), we included a total of 12 studies in the meta-analyses. Six studies were excluded because they lacked estimates of mean 25(OH)D levels, prevalence of vitamin D status (25(OH)D < 50 or < 75 nmol/L) or did not report estimates from children aged between 0 and 8 years. The meta-analyses included 2128 children from five African countries with mean ages ranging from one to 47 months and included estimates from healthy children in 9 case-control and three population-based studies in addition to the current study. Overall, mean 25(OH)D level in young African children was 73.2 nmol/L (95% CI 66.4, 80.1) (Fig. 2) and prevalence of 25(OH)D levels < 50 and < 75 nmol/L was 10.9% (95% CI 6.9, 15.5) and 49.1% (95% CI 40.8, 57.5), respectively (Additional file 12: Figure S4 and Additional file 13: Figure S5). Only a single eligible study reported prevalence defined by 25(OH)D levels < 30 nmol/L [4] precluding meta-analysis. There was high heterogeneity between studies included in the meta-analyses with overall *I*^2^ ranging from 95.1% and 99.4% (*p* < 0.01).

## Discussion

Little is known about the vitamin D status of African children. In this study, overall median 25(OH)D level was 77.6 nmol/L (IQR 63.6, 94.2) and prevalence of 25(OH)D cut-offs between 50 and 75, < 50, and < 30 nmol/L was 37.1%, 7.8%, and 0.6%, respectively, in young African children. The prevalence of vitamin D deficiency (25(OH)D levels < 50 nmol/L) was higher in South African children and during the South African winter or the long rainy season in East Africa. Median 25(OH)D levels decreased with increasing age and with inflammation but did not differ by sex or nutritional status after adjustment for potential confounders in multivariable models. Malaria parasitaemia was associated with lower 25(OH)D levels overall and in Kenyan children, but not in Ugandan, Burkinabe or Gambian children. The most common Gc variant was Gc1f (83.3%), followed by Gc1s (8.5%) and Gc2 (8.2%), with Gc2 being associated with the lowest 25(OH)D levels and the highest prevalence of low vitamin D status (25(OH)D levels < 50 and 50–75 nmol/L). In a meta-analysis of the current and previous studies of young African children overall mean 25(OH)D level was 73.2 nmol/L.

Our median 25(OH)D estimate of 77.6 nmol/L is comparable to values reported in previous studies of healthy young children in sub-Saharan Africa including reported levels of 80.4 nmol/L in Uganda, 78.6 nmol/L in Malawi, and 72.4 nmol/L in Nigeria (Fig. 2). However, studies in 198 Nigerian children and 581 Tanzanian infants living in urban areas reported lower mean levels of 64.8 and 64.9 nmol/L, respectively [8, 9]. The prevalence of vitamin D deficiency (25(OH)D levels < 50 nmol/L) was 7.8% overall, with a higher prevalence of 13.5% in South Africa. Previous studies of healthy young children have reported higher prevalence estimates of 13.6% in Kenya, 15.0% in Uganda, and 25.8% in Nigeria [4, 8, 15]. Prevalence estimates were also higher in young children from other continents, including estimates of 15% in the USA, 14% in Japan, and 11% in China [34–36]. Vitamin D deficiency is also more prevalent in northern African countries [2], and the higher vitamin D status observed in our study might be explained by differences in latitude, geography, skin pigmentation, clothing coverage, and religious and cultural practices across Africa [37]. The year-round abundance of sunshine in sub-Saharan Africa may also explain higher vitamin D status although vitamin D supplementation and food fortification is less common. Since vitamin D deficient rickets is rare at levels above 30 nmol/L, the very low prevalence of 25(OH)D levels < 30 nmol/l (0.6%) in the current study suggests that rickets in African children is more likely to be caused by calcium rather than vitamin D deficiency. Conversely, we found very few children with 25(OH)D levels above 150 nmol/L suggesting that even plenty of exposure to sunlight rarely generates these high levels probably because vitamin D production in the skin is highly regulated [1].

In the present study, 25(OH)D levels decreased consistently with age overall and in all countries except The Gambia (Additional file 3: Table S2), perhaps because of older age in the Gambian children and corresponding cultural habits. Age explained 4.5% of the variation in 25(OH)D levels. Previous studies of school children (aged 5–18 years) from South Africa, Ethiopia and Algeria have also reported that 25(OH)D levels decreased with age [11, 38, 39]. However, a single study in Malawian pre-school children reported an increase in 25(OH)D levels with age; however, this study was small (*n*=21) and included infants of mothers living with HIV in Malawi [6]. Studies from high-income countries have reported an increase in 25(OH)D levels with age, but children in these studies received vitamin D supplementation or fortification [34, 35, 40]. In a meta-analysis of previous studies from Africa, children had higher vitamin D status than adults in African populations [2] possibly due to increased time spent outdoors.

In addition, we found limited evidence of an association between 25(OH)D levels and sex, although overall girls had a 32% (95% CI 1.04, 1.68) increased risk of vitamin D deficiency (25(OH)D < 50 nmol/L) compared to boys. Similar studies from South Africa, China, and Ecuador have not found sex-related differences in 25(OH)D levels [36, 38, 41]. We also found evidence of seasonality in 25(OH)D levels with the strongest effect in South Africa, and more variable effects observed across the sub-Saharan African countries during the rainy season. These findings may be explained by colder winters in South Africa with greater coverage of skin by clothing and more time spent indoors and in East Africa by increased cloud cover and less time spent outdoors during the long rains. Season explained 3.8% of the variation in 25(OH)D levels. These findings agree with previous studies that evaluated the effect of seasonality in vitamin D status in Africa, although many of these studies were in South Africa or northern African countries [2].

We did not find associations between 25(OH)D levels and stunting, underweight, or wasting in adjusted regression analyses. Similarly, studies in young children from Tanzania (*n* = 948) and Nepal (*n* = 280) reported that 25(OH)D levels were not associated with stunting, underweight or wasting [10, 42]. However, severe wasting was associated with 25(OH)D levels < 30 nmol/L in 21 Kenyan children with rickets [4]. In addition, Mokhtar and colleagues reported that low 25(OH)D levels (< 42.5 nmol/L) were more common among stunted and underweight Ecuadorian children [41]. The lack of association between vitamin D status and anthropometric indices in the current study suggests that sunlight may be more important than dietary intake in influencing vitamin D status in Africa.

We observed a positive correlation between 25(OH)D levels and markers of inflammation overall and children with inflammation had higher 25(OH)D levels than those without after adjusting for potential confounders. Our findings agree with results from a small case-control study in Egyptian children with sepsis (*n* = 40, mean age 6 years) and a large community-based study of children (*n* = 4274, mean age 9.9 years) in England, which reported that 25(OH)D levels increased with increasing levels of CRP and IL-6 [7, 13]. In contrast, a recent meta-analysis of 24 randomised controlled trials reported that vitamin D supplementation had an overall effect of reducing IL6, but not CRP or other inflammatory markers, although many trial participants had medical conditions [14]. A nationally representative survey of 15,167 adults in the USA reported an inverse association between 25(OH)D and CRP levels at 25(OH)D levels < 52 nmol/L and a positive association above this level [43], suggesting that there may be a U-shaped association, which might explain previously mixed findings. In our study, 90% of children had 25(OH)D levels > 52 nmol/L, perhaps explaining the positive association observed between inflammation and 25(OH)D levels. However, the association between inflammation and 25(OH)D may not be clinically relevant since inflammation explained only 1.2% of the observed variation in 25(OH)D levels.

In this study, 25(OH)D levels between 50 and 75 nmol/L were associated with an increased risk of afebrile malaria parasitaemia overall compared to levels > 75 nmol/L. In contrast, a study in western Kenya found no association between 25(OH)D levels and malaria parasitaemia in newborns and their mothers [44]. However, another study in Uganda reported that children with severe malaria had lower 25(OH)D levels than healthy community children [15]. Malaria explained only 0.4% of the variation in 25(OH)D levels in our study, suggesting that this association may not be clinically significant. No clinical trials have yet investigated the effect of vitamin D supplementation on malaria incidence or treatment outcomes [45].

In the current study, we found that Gc1f was the most frequent Gc variant (83.3%) and the Gc1s and Gc2 variants were less frequent (8.5 and 8.2%, respectively). The Gc1f and Gc1s variants were associated with higher 25(OH)D levels compared to the Gc2 variant. In agreement with our study, a study involving 237 Gambian children with similar Gc variant frequencies (Gc1f was 86%, Gc1s 11% and Gc2 3%) reported that Gc1f was associated with higher 25(OH)D levels than the other haplotypes combined [19]. Gc1f, the most frequent Gc variant in Africans, has a higher binding affinity for vitamin D compared to the Gc1s and Gc2 variants which are more frequent in Europeans and Asians [16, 18]. In a study involving adults from The Gambia and the UK (*n*=36), Gc1f was associated with higher total 25(OH)D levels and shorter 25(OH)D half-life [20]. In addition, DBP levels and genetic polymorphisms have been linked to lower levels of 25(OH)D in black American adults compared with white American adults [17]. In another study involving multi-ethnic children, differences in DBP polymorphisms were associated with lower vitamin D status in African children and reduced response to vitamin D intake compared to Hispanic and Caucasian children [46]. The differences in 25(OH)D levels attributed to DBP polymorphism has led to the suggestion that DBP variants and levels should be considered in the assessment of vitamin D status in different populations [47].

### Strengths and limitations

To the best of our knowledge, the current study, which included a total of 4509 children, is the largest study to date to evaluate vitamin D status and its predictors in young African children. The study further evaluated the effect of common genetic polymorphisms encoding the vitamin D binding protein on 25(OH)D levels. However, our findings should be interpreted in the context of some limitations. First, due to the cross-sectional nature of our study, we could not evaluate temporal changes in 25(OH)D and CRP levels or infer the direction of causality for the observed associations. We also did not measure vitamin D binding protein, parathyroid hormone, calcium levels, dietary or supplementary vitamin D intake, or exposure to sunlight, factors which have been shown to influence vitamin D status. DBP may also be a better marker of the effect of inflammation since it is reduced during tissue damage and in inflammation resulting in the reduction of circulating 25(OH)D levels and other vitamin D metabolites [48]. Our study cohorts were also diverse with different ethnicities and ages, and hence may not be generalisable to other age groups or African countries, although cohort-specific data and analyses were presented.

## Conclusions

Approximately 0.6% and 7.8% of children in our study had 25(OH)D levels of < 50 or < 30 nmol/L, and approximately one third of children had 25(OH)D levels between 50 and 75 nmol/L. Our data indicate that older children and those who live further from the equator may be at a higher risk of vitamin D deficiency. Stunting, underweight and wasting were not associated with vitamin D deficiency suggesting that sunlight is a more important source of vitamin D than dietary intake in children living in sub-Saharan Africa. Genetic differences in DBP also altered 25(OH)D levels. Further research is required to understand the effects of inflammation and malaria on vitamin D status in Africa and to investigate the causality between vitamin D status and infectious diseases like malaria, which are common in African children. These findings may have important implications for public health strategies involving young children in Africa.

## Supplementary Information

**Additional file 1: Supplementary methods.** (Genotyping and SNP quality control). This file describes the methods used in genotyping and SNP quality control, QC parameters, allocation of vitamin D binding protein Gc variants and haplotypes.

**Additional file 2: Table S1.** Databases search keywords. This table includes keywords used systematically search PubMed and Embase for articles of studies that measured vitamin D status in young children.

**Additional file 3: Table S2.** Median 25(OH)D levels by study variable in each country. This is a table of median 25(OH)D levels for each study variable in each country.

**Additional file 4: Table S3.** Multivariable logistic regression analyses of factors associated with low vitamin D status in each country. This is a table showing odd ratios (with their corresponding 95% CIs and p values) from multivariable logistic regression analyses.

**Additional file 5: Table S4.** Univariable linear regression of 25(OH)D concentrations by study variables in each country. This is a table of univariable linear regression coefficients (with 95% CI) and p values presented by country.

**Additional file 6: Table S5.** Multivariable regression of 25(OH)D concentrations by study variables in each country. This is a table of multivariable linear regression coefficients (with 95% CI) and p values presented by country.

**Additional file 7: Table S6.** Median 25(OH)D levels by vitamin D binding protein haplotype and Gc variant in each country. This is a table of DBP variant and haplotype frequencies and median 25(OH)D levels by country.

**Additional file 8: Table S7.** Summary characteristics of studies that evaluated the vitamin D status of African children (in alphabetical order). This is a table describing characteristics of past studies that evaluated the vitamin D status of young children in Africa.

**Additional file 9: Figure S1.** The precision profile of the 25(OH)D assay. This is a scatter plot of coefficient of variation (%) of the 25(OH)D assay used in this study.

**Additional file 10: Figure S2.** Boxplots of 25(OH)D concentrations by country (A), age categories (B), sex (C), season (D) stunting (E), underweight (F), wasting (G), inflammation (H), malaria (I), vitamin D binding protein (DBP) isotype (J) and Gc variant (K). This is a grid of boxplots of 25(OH)D concentrations for each study variable.

**Additional file 11: Figure S3.** Boxplots of CRP levels overall and by country. These are boxplots of CRP levels.

**Additional file 12: Figure S4.** Meta-analysis of studies that estimated the prevalence of 25(OH)D levels < 50 nmol/L in healthy children aged 0–8 years in Africa. This is a meta-analysis plot of prevalence estimates of vitamin D deficiency (defined by 25(OH)D levels < 50 nmol/L) in young children included in the current and previous studies.

**Additional file 13: Figure S5.** Meta-analysis of studies that estimated the prevalence of 25(OH)D levels < 75 nmol/L in healthy children aged 0–8 years in Africa. This is a meta-analysis plot of prevalence estimates of low vitamin D status (defined by 25(OH)D levels < 75 nmol/L) in young children included in the current and previous studies.

## Data Availability

The data and analyses scripts underlying this article are available in Harvard Dataverse at 10.7910/DVN/MH1LPE and applications for data access can be made through the Kilifi Data Governance Committee cgmrc@kemri-wellcome.org.

## Abbreviations

25(OH)D: 25-hydroxyvitamin D
CRP: C-reactive protein
EMaBS: Entebbe Mother and Baby Study
HAZ: Height-for-age *z*-scores
WAZ: Weight-for-age *z*-scores
WHZ: Weight-for-height *z*-scores
DBP: Vitamin D binding protein
