# Supplementary material for: Prevalence and predictors of vitamin D deficiency in young African children
Source: BMC Med. 2021 May 20;19:115. doi: 10.1186/s12916-021-01985-8 (PMC8136043; doi:10.1186/s12916-021-01985-8)
Supplement: Supplementary file 1 — Additional file 1: Supplementary methods. (Genotyping and SNP quality control). This file describes the methods used in genotyping and SNP quality control, QC parameters, allocation of vitamin D binding protein Gc variants and haplotypes. [file 12916_2021_1985_MOESM1_ESM.docx]

**Supplementary methods (Genotyping and SNP quality control)**

Genotyping of samples from Uganda, South Africa and Burkina Faso were performed using Illumina HumanOmni 2.5 M-8 (“octo”) BeadChip array version 1.1 (Illumina Inc., San Diego, USA) by Genotyping Core facilities at the Wellcome Trust Sanger Institute, and from Kenya and The Gambia using the H3A Custom Genotyping Array (H3Africa v1.0) by Oxford Genomics Centre at the Wellcome Centre for Human Genetics. Genotypes were called from intensities using two clustering algorithms (Illuminus and GenCall) in GenomeStudio (Illumina Inc., San Diego, USA). SNP quality control (QC) was performed separately for each cohort using the H3Africa GWAS pipeline version 3 (<https://github.com/h3abionet/h3agwas>). Samples that were filtered during the QC included samples with call rate less than 97%, heterozygosity >3 SDs around the mean, sex discrepancy (genotype and allocated sex differed), from related individuals (identity by descent >0.9). The following SNPs were filtered from the remaining sample: SNPs with <97% call rate, minor allele frequency <0.01 and Hardy–Weinberg equilibrium (HWE) <0.008. Imputation of missing genotypes was performed using TOPMED imputation server (<https://imputation.biodatacatalyst.nhlbi.nih.gov/>). The single nucleotide polymorphisms rs7041 and rs4588 genotypes were retrieved from imputed data using qctool (<https://www.well.ox.ac.uk/~gav/qctool_v1/>) and their combination (rs7041 and rs4588, respectively) used to allocate DBP haplotypes (rs7041, rs4588) as follows: Gc1f/f (TT, CC), Gc1f/s (TG, CC), Gc1f/2 (TT, CA), Gc1s/s (GG, CC), Gc1s/2 (TG, CA), and Gc2/2 (TT, AA) (Table 2). The DBP alleles were used to determine the Gc variants including Gc1f, Gc1s and Gc2, as previously described [1, 2].
