# Supplementary material for: Prevalence and predictors of vitamin D deficiency in young African children
Source: BMC Med. 2021 May 20;19:115. doi: 10.1186/s12916-021-01985-8 (PMC8136043; doi:10.1186/s12916-021-01985-8)
Supplement: Supplementary file 2 — Additional file 2: Table S1. Databases search keywords. This table includes keywords used systematically search PubMed and Embase for articles of studies that measured vitamin D status in young children. [file 12916_2021_1985_MOESM2_ESM.docx]

**Table S1. Database search keywords**

| **Database** | **Search terms** |
| --- | --- |
| PubMed | ("Vitamin D"[Mesh] OR "Vitamin D Deficiency"[Mesh] OR "Cholecalciferol"[Mesh] OR "25-Hydroxyvitamin D 2"[Mesh] OR "Calcifediol"[Mesh] OR "Ergocalciferols"[Mesh]) AND ("Child"[Mesh] OR "Child, Preschool"[Mesh] OR "Infant"[Mesh]) NOT "Infant, Newborn"[Mesh] AND ("African Continental Ancestry Group"[Mesh] OR “Algeria”[Mesh] OR “Egypt”[Mesh] OR “Libya”[Mesh] OR “Morocco”[Mesh] OR “South Sudan”[Mesh] OR “Sudan”[Mesh] OR “Tunisia”[Mesh] OR “Burundi”[Mesh] OR “Comoros”[Mesh] OR “Djibouti”[Mesh] OR “Eritrea”[Mesh] OR “Ethiopia”[Mesh] OR “Kenya”[Mesh] OR “Madagascar”[Mesh] OR “Malawi”[Mesh] OR “Mauritius”[Mesh] OR “Comoros”[Mesh] OR “Mozambique”[Mesh] OR “Reunion”[Mesh] OR “Rwanda”[Mesh] OR “Seychelles”[Mesh] OR “Somalia”[Mesh] OR “Tanzania”[Mesh] OR “Uganda”[Mesh] OR “Zambia”[Mesh] OR “Zimbabwe”[Mesh] OR “Benin”[Mesh] OR “Burkina Faso”[Mesh] OR “Cabo Verde”[Mesh] OR “Cote d'Ivoire”[Mesh] OR “Gambia”[Mesh] OR “Ghana”[Mesh] OR “Guinea”[Mesh] OR “Guinea-Bissau”[Mesh] OR “Liberia”[Mesh] OR OR “Mali”[Mesh] OR “Mauritania”[Mesh] OR “Niger”[Mesh] OR “Nigeria”[Mesh] OR “Atlantic Islands”[Mesh] OR “Senegal”[Mesh] OR “Sierra Leone”[Mesh] OR “Togo”[Mesh] OR “Angola”[Mesh] OR “Cameroon”[Mesh] OR “Central African Republic”[Mesh] OR “Chad”[Mesh] OR “Congo”[Mesh] OR “Democratic Republic of the Congo”[Mesh] OR “Equatorial Guinea”[Mesh] OR “Gabon”[Mesh] OR “Sao Tome and Principe”[Mesh] OR “Botswana”[Mesh] OR “Lesotho”[Mesh] OR “Namibia”[Mesh] OR “South Africa”[Mesh] OR “Eswatini”[Mesh]) |
| Embase | ( (" vitamin D" and ("Child" OR "Child, Preschool" OR "Infant") NOT "Newborn" and ("Algeria" or " Egypt" or " Libya" or " Morocco" or " South Sudan" or " Sudan" or " Tunisia" or " Western Sahara" or " Burundi" or " Comoros" or " Djibouti" or " Eritrea" or " Ethiopia" or " Kenya" or " Madagascar" or " Malawi" or " Mauritius" or " Comoros" or " Mozambique" or " Reunion" or " Rwanda" or " Seychelles" or " Somalia" or " Tanzania" or " Uganda" or " Zambia" or " Zimbabwe" or " Benin" or " Burkina Faso" or " Cape Verde" or " Cote d'Ivoire " or " Ivory Coast" or " Gambia" or " Ghana" or " Guinea" or " Guinea-Bissau" or " Liberia" or " Mali" or " Mauritania" or " Niger" or " Nigeria" or "Atlantic Islands" or " Senegal" or " Sierra Leone" or " Togo" or " Angola" or " Cameroon" or " Central African Republic" or " Chad" or " Congo" or " Democratic Republic of the Congo" or " Equatorial Guinea" or " Gabon" or " Sao Tome and Principe" or " Botswana" or " Lesotho" or " Namibia" or " South Africa" or " Eswatini")) not "African American"). |
