# Supplementary material for: Prevalence and predictors of vitamin D deficiency in young African children
Source: BMC Med. 2021 May 20;19:115. doi: 10.1186/s12916-021-01985-8 (PMC8136043; doi:10.1186/s12916-021-01985-8)
Supplement: Supplementary file 3 — Additional file 3: Table S2. Median 25(OH)D levels by study variable in each country. This is a table of median 25(OH)D levels for each study variable in each country. [file 12916_2021_1985_MOESM3_ESM.docx]

**Table S2. Median 25(OH)D levels by study variable in each country**

|  | **Kenya** | | **Uganda** | | | **Burkina Faso** | | | **The Gambia** | | | **South Africa** | | |  |
| --- | --- | --- | --- | --- | --- | --- | --- | --- | --- | --- | --- | --- | --- | --- | --- |
|  | **Median (IQR) 25(OH)D nmol/L** | **P** | | **Median (IQR) 25(OH)D nmol/L** | **P** | | **Median (IQR) 25(OH)D nmol/L** | **P** | | **Median (IQR) 25(OH)D nmol/L** | **P** | | **Median (IQR) 25(OH)D nmol/L** | **P** | |
| **Overall** | 81.0 (66.3, 101.6) |  | | 78.6 (65.1, 94.5) |  | | 78.4 (64.5, 91.3) |  | | 71.2 (59.1, 84.2) | 0.0001 | | 76.2 (60.6, 91.9) |  | |
| **Age categories (months)** |  |  | |  |  | |  |  | |  |  | |  |  | |
| < 12 | 101.8 (82.7, 121.8) | 0.0001 | | 97.7 (80.3, 115.9) | 0.0003 | | 103.8 (80.2, 120.5) | 0.0001 | | n/a | 0.32 | | 78.3 (63, 94.4) | 0.003 | |
| 12 -24 | 87.7 (72.6, 104.3) |  | | 79.5 (66.1, 96.2) |  | | 82.7 (70.1, 96.1) |  | | 73.4 (60.3, 93.0) |  | | 73.9 (58.2, 89.9) |  | |
| 24-36 | 71.1 (58.1, 87.4) |  | | 78.9 (65.0, 93.0) |  | | 72.0 (61.4, 82.0) |  | | 69.8 (59.3, 82.7) |  | | 61.8 |  | |
| 36-48 | 68.0 (57.6, 79.0) |  | | 76.5 (62.8, 96.7) |  | | n/a |  | | 70.4 (57.2, 80.5) |  | | n/a |  | |
| 48+ | 67.0 (54.8, 78.0) |  | | 73.4 (59.9, 86.2) |  | | n/a |  | | 73.0 (62.0, 85.8) |  | | n/a |  | |
| **Sex** |  |  | |  |  | |  |  | |  |  | |  |  | |
| Males | 82.2 (67.4, 101.6) | 0.25 | | 78.5 (65.5, 94.9) | 0.71 | | 76.8 (62.8, 92.2) | 0.33 | | 72.8 (62.1, 86.0) | 0.038 | | 76.8 (60.9, 92.1) | 0.36 | |
| Females | 80.3 (65.0, 101.6) |  | | 78.7 (64.8, 94.5) |  | | 80.2 (67.0, 90.6) |  | | 69.6 (57.2, 82.0) |  | | 74.6 (60.3, 91.5) |  | |
| **Season^#^** |  |  | |  |  | |  |  | |  |  | |  |  | |
| Summer/short rains/dry | 77.2 (62.9, 98.8) | 0.04 | | 79.1 (67.0, 94.8) | 0.0002 | | 76.6 (62.4, 92.3) | 0.01 | | - | 0.86 | | 80.2 (63.8, 96.1) | 0.0001 | |
| Autumn/dry | 82.2 (66.8, 102.3) |  | | 83.0 (66.8, 96.8) |  | | 84.1 (68.4, 96.2) |  | | - |  | | 88.8 (71.4, 103.0) |  | |
| Winter/long rains | 87.8 (70.4, 106.4) |  | | 74.7 (62.5, 87.3) |  | | 74.6 (64.3, 85.5) |  | | 71.6 (59.1, 84.4) |  | | 69.3 (53.2, 85.7) |  | |
| Spring/dry | 80.5 (71.9, 97.5) |  | | 79.1 (64.8, 96.8) |  | | 72.3 (69.1, 85.0) |  | | 69.0 (60.3, 82.8) |  | | 72.6 (60.1, 87.8) |  | |
| **Nutritional status** |  |  | |  |  | |  |  | |  |  | |  |  | |
| Not stunted^†^ | 71.0 (57.2, 89.0) | 0.76 | | 78.5 (65.4, 94.5) | 0.89 | | 78.8 (64.0, 89.7) | 0.72 | | 72.3 (60.2, 85.0) | 0.15 | | n/a | n/a | |
| Stunted | 70.0 (58.2, 80.0) |  | | 78.9 (64.1, 95.1) |  | | 75.4 (62.9, 91.3) |  | | 69.2 (56.8, 84.1) |  | | n/a | n/a | |
| Not underweight**^‡^** | 79.0 (65.6, 96.0) | 0.28 | | 78.6 (65.2, 94.8) | 0.82 | | 77.8 (64.3, 90.3) | 0.83 | | 70.5 (58.2, 83.8) | 0.37 | | n/a | n/a | |
| Underweight | 76.4 (62.0, 94.7) |  | | 78.5 (63.3, 91.9) |  | | 75.8 (61.1, 93.0) |  | | 72.0 (60.2, 85.3) |  | | n/a | n/a | |
| Not wasted**^§^** | 70.6 (58.0, 85.9) | 0.67 | | 78.5 (65.2, 94.4) | 0.19 | | 77.5 (63.9, 90.5) | 0.68 | | 70.1 (57.6, 83.2) | 0.002 | | n/a | n/a | |
| Wasted | 69.0 (59.6, 79.4) |  | | 83.9 (68.2, 101.2) |  | | 79.8 (62.3, 100.6) |  | | 80.3 (68.0, 89.4) |  | | n/a | n/a | |
| **Inflammation**^¶^ |  |  | |  |  | |  |  | |  |  | |  |  | |
| Without inflammation | 81.0 (66.0, 100.1) | 0.094 | | 77.4 (64.2, 92.4) | 0.0003 | | 78.4 (63.6, 90.6) | 0.41 | | 69.8 (58.1, 82.1) | <0.0001 | | 74.9 (59.3, 90.7) | 0.0001 | |
| With inflammation | 82.4 (68.0, 106.9) |  | | 83.0 (68.8, 98.1) |  | | 78.2 (65.8, 93.2) |  | | 81.1 (69.3, 94.8) |  | | 83.4 (67.9, 98.4) |  | |
| **Malaria**^†^ |  |  | |  |  | |  |  | |  |  | |  |  | |
| Without malaria | 80.2 (66.0, 97.0) | <0.0001 | | 78.4 (65.0, 94.4) | 0.18 | | 77.8 (64.0, 90.5) | 0.24 | | 71.3 (59.5, 84.4) | 0.68 | | n/a |  | |
| With malaria | 67.7 (57.8, 79.6) |  | | 82.3 (67.1, 96.8) |  | | 74.8 (60.8, 87.1) |  | | 70.8 (58.1, 80.7) |  | | n/a |  | |
| ^*^P values were obtained by performing a Wilcoxon rank-sum test for variables with two categories or Kruskal-Wallis equality-of-populations rank test for variables with more than one. **^#^**Season was based on 3 monthly intervals. In South Africa the seasons are summer, autumn, winter and spring, in Uganda and Kenya there are two rainy seasons and in Burkina Faso and The Gambia there is a single rainy season although timing of the rains is often unpredictable and may vary from the times shown here. 1^st^ season as December-February; 2^nd^ season as March-May; 3^rd^ season as June-August; 4^th^ season as September-November. ^†^Stunting was defined as height-for-age Z score <-2; **^‡^**underweight as weight-for-age Z score <-2; **^§^** wasting as weight-for-height Z score < -2; ^¶^inflammation as CRP >5 mg/L or ACT >0.6 g/L (ACT, but not CRP was available for The Gambia); and ^f^malaria as presence of *P. falciparum* parasites on blood film. | | | | | | | | | | | | | | |  |
