# Supplementary material for: Prevalence and predictors of vitamin D deficiency in young African children
Source: BMC Med. 2021 May 20;19:115. doi: 10.1186/s12916-021-01985-8 (PMC8136043; doi:10.1186/s12916-021-01985-8)
Supplement: Supplementary file 4 — Additional file 4: Table S3. Multivariable logistic regression analyses of factors associated with low vitamin D status in each country. This is a table showing odd ratios (with their corresponding 95% CIs and p values) from multivariable logistic regression analyses. [file 12916_2021_1985_MOESM4_ESM.docx]

**Table S3. Multivariable logistic regression analyses of factors associated with low vitamin D status**

|  |  | **Overall** | | **Kenya** | | **Uganda** | | **Burkina Faso** | | **The Gambia** | | | **South Africa** | |  |
| --- | --- | --- | --- | --- | --- | --- | --- | --- | --- | --- | --- | --- | --- | --- | --- |
|  |  | **OR (95% CI)** | **P** | **OR (95% CI)** | **P** | **OR (95% CI)** | **P** | **OR (95% CI)** | **P** | | **OR (95% CI)** | **P** | **OR (95% CI)** | **P** |  |
| **Age (years)** | **25(OH)D 50–75**  **25(OH)D <50** | 1.43 (1.34, 1.52)  1.69 (1.52, 1.89) | <0.0001  <0.0001 | 1.67 (1.53, 1.83)  1.95 (1.69, 2.26) | <0.0001  <0.0001 | 1.24 (1.07, 1.44)  1.57 (1.20. 2.06) | 0.003  0.001 | 3.50 (1.75, 6.98)  2.49 (0.61, 10.03) | 0.0004  0.20 | | 0.91 (0.79, 1.05)  0.96 (0.74, 1.25) | 0.21  0.87 | 4.45 (0.81, 24.60)  0.93 (0.04, 23.05) | 0.087  0.96 |  |
| **Sex: female** | **25(OH)D 50–75**  **25(OH)D <50** | 1.09 (0.97, 1.25)  1.32 (1.05, 1.68) | 0.15  0.021 | 1.31 (1.02, 1.67)  1.23 (0.74, 2.05) | 0.033  0.43 | 0.97 (0.77, 1.22)  1.05 (0.63, 1.76) | 0.79  0.85 | 0.70 (0.43, 1.14)  0.96 (0.35, 2.64) | 0.15  0.93 | | 1.07 (0.76, 1.48)  2.27 (1.27, 4.06) | 0.75  0.006 | 1.28 (0.95, 1.73)  1.31 (0.86, 2.00) | 0.10  0.21 |  |
| **Season^#^** | **25(OH)D 50–75**  Summer/short rains/dry  Autumn/dry  Winter/long rains  Spring/dry  **25(OH)D <50**  Summer/short rains/dry  Autumn/dry  Winter/long rains  Spring/dry | Ref  0.79 (0.65, 0.96)  1.34 (1.09, 1.65)  1.13 (0.91, 1.40)  Ref  0.65 (0.43, 0.96)  2.55 (1.75, 3.72)  1.60 (1.07, 2.38) | 0.016  0.005  0.26  -  0.032  <0.00010.022 | Ref  0.88 (0.64, 1.22)  0.87 (0.47, 1.63)  1.03 (0.60, 1.79)  Ref  0.75 (0.40, 1.40)  3.21 (1.27, 8.14)  0.24 (0.03, 1.87) | -  0.45  0.67  0.89  -  0.37  0.014  0.17 | Ref  0.82 (0.58, 1.15)  1.42 (1.03, 1.95)  0.84 (0.61, 1.17)  Ref  0.78 (0.30, 1.98)  2.49 (1.17, 5.33)  2.08 (0.99, 4.38) | -  0.24  0.033  0.30  0.60  0.019  0.053 | Ref  0.58 (0.30, 1.14)  0.78 (0.37, 1.64)  3.27 (0.49, 21.65)  Ref  0.10 (0.02, 0.55)  0.43 (0.11, 1.70)  ** | -  0.11  0.51  0.22  0.007 0.23  - | | -  -  Ref  1.21 (0.76, 1.93)  -  -  Ref  0.56 (0.18, 1.70) | -  -  -  0.43  -  -  -  0.30 | Ref  0.58 (0.35, 0.97)  1.95 (1.28, 2.98)  1.73 (1.14, 2.64)  Ref  0.60 (0.25, 1.44)  3.92 (2.07, 7.42)  2.46 (1.28, 4.71) | -  0.036  0.002  0.010  0.25  <0.0001  0.007 |  |
| **Stunted^*^** | **25(OH)D 50–75**  **25(OH)D <50** | 1.25 (1.01, 1.54)  1.05 (0.69, 1.58) | 0.038  0.82 | 1.45 (0.77, 2.71)  0.53 (0.16, 1.81) | 0.25  0.72 | 1.16 (0.84, 1.61)  0.91 (0.43, 1.94) | 0.36  0.82 | 1.13 (0.67, 1.93)  0.83 (0.26, 2.64) | 0.65  0.75 | | 1.19 (0.79, 1.78)  1.16 (0.59, 2.29) | 0.41  0.68 | n/a  n/a | n/a  n/a |  |
| **Underweight^†^** | **25(OH)D 50–75**  **25(OH)D <50** | 1.05 (0.82, 1.32)  0.88 (0.54, 1.41) | 0.71  0.58 | 0.87 (0.51, 1.49)  0.96 (0.33, 2.79) | 0.58  0.95 | 1.35 (0.89, 2.07)  0.40 (0.09, 1.69) | 0.16  0.21 | 1.07 (0.57, 2.01)  0.55 (0.11, 2.64) | 0.83  0.45 | | 0.84 (0.55, 1.30)  0.95 (0.44, 2.03) | 0.44  0.89 | n/a  n/a | n/a  n/a |  |
| **Wasted**^‡^ | **25(OH)D 50–75**  **25(OH)D <50** | 0.69 (0.48, 1.00)  0.63 (0.29, 1.38) | 0.049  0.25 | 1.51 (0.56, 4.07)  0.91 (0.09, 9.77) | 0.42  0.94 | 0.71 (0.39, 1.29)  0.81 (0.24, 2.75) | 0.26  0.73 | 0.55 (0.18, 1.68)  1.38 (0.24, 7.97) | 0.29  0.72 | | 0.51 (0.26, 1.00)  0.19 (0.02, 1.45) | 0.047  0.11 | n/a  n/a | n/a  n/a |  |
| **Inflammation^§^** | **25(OH)D 50–75**  **25(OH)D <50** | 0.74 (0.64, 0.87)  0.58 (0.43, 0.81) | 0.0002  0.001 | 0.86 (0.65, 1.15)  0.66 (0.36, 1.21) | 0.31  0.18 | 0.65 (0.49, 0.86)  0.78 (0.42, 1.45) | 0.003  0.36 | 0.84 (0.50, 1.42)  2.27 (0.81, 6.40) | 0.52  0.12 | | 0.57 (0.36, 0.93)  ** | 0.023  0 | 0.68 (0.46, 1.01)  0.40 (0.21, 0.77) | 0.059  0.006 |  |
| **Malaria^††^** | **25(OH)D 50–75**  **25(OH)D <50** | 1.48 (1.18, 1.88)  1.02 (0.65, 1.61) | 0.001  0.93 | 1.75 (1.19, 2.57)  0.94 (0.47, 1.89) | 0.005  0.87 | 1.10 (0.69, 1.77)  0.41 (0.10, 1.78) | 0.68  0.24 | 1.20 (0.63, 2.28)  1.26 (0.37, 4.35) | 0.59  0.71 | | 1.32 (0.75, 2.30)  0.82 (0.29, 2.36) | 0.34  0.72 | n/a  n/a | n/a  n/a |  |
| **Gc variant**^‡^ | **25(OH)D 50–75**  Gc1f  Gc1s  Gc2  **25(OH)D <50**  Gc1f  Gc1s  Gc2 | Ref  0.93 (0.78, 1.11)  1.62 (1.36, 1.93)  Ref  0.73 (0.52, 1.06)  1.69 (1.23, 2.31) | -  0.45  <0.0001  -  0.096  0.001 | Ref  0.96(0.65, 1.43)  1.55 (1.10, 2.20)  Ref  1.38 (0.67, 2.87)  3.57 (1.97, 6.46) | -  0.85  0.013  -  0.39  <0.0001 | Ref  0.96 (0.71, 1.29)  1.95 (1.49, 2.55)  Ref  0.48 (0.20, 1.11)  1.28 (0.70, 2.32) | -  0.78  <0.0001  -  0.087  0.43 | Ref  0.58 (0.32, 1.05)  1.13 (0.51, 2.51)  Ref  1.38 (0.08, 1.67)  1.01 (0.19, 5.27) | 0.071  0.77  0.20  0.99 | | Ref  0.92 (0.61, 1.39)  1.13 (0.55, 2.31)  Ref  0.43 (0.17, 1.09)  1.84 (0.63, 5.35) | -  0.70  0.74  0.075  0.26 | Ref  1.12(0.71, 1.76)  2.31 (0.64, 8.31)  Ref  1.04 (0.53, 2.03)  1.37 (0.72, 2.60) | -  0.62  0.15  -  0.92  0.34 |  |
| This is a logistic regression with variables as exposures and vitamin D status as a binary outcome (>75 and 50–75 or <50 nmol/L). **^#^**Season was based on 3 monthly intervals (in South Africa the seasons are summer, autumn, winter and spring, in Uganda and Kenya there are two rainy seasons and in Burkina Faso and The Gambia there is a single rainy season).**^*^**Stunted was defined as height-for-age Z score < -2; **^†^**underweight as weight-for-age Z score < -2; ^‡^wasted as weight-for-height Z score < -2;**^§^**inflammation was defined as CRP >5 mg/L or ACT >0.6 g/L (ACT only was available for The Gambia); **^††^**malaria as presence of *P. falciparum* parasites on blood film. Covariates in the regression models included age, sex, season and inflammation and for the overall model, study site. Anthropometric measurements were not available for South African children and they were not exposed to malaria. OR, odds ratio; CRP, C-reactive protein. ** this model couldn’t run since all children with 25(OHD <50 nmol/L had inflammation. | | | | | | | | | | | | | | | |
