# Supplementary material for: Prevalence and predictors of vitamin D deficiency in young African children
Source: BMC Med. 2021 May 20;19:115. doi: 10.1186/s12916-021-01985-8 (PMC8136043; doi:10.1186/s12916-021-01985-8)
Supplement: Supplementary file 5 — Additional file 5: Table S4. Univariable linear regression of 25(OH)D concentrations by study variables in each country. This is a table of univariable linear regression coefficients (with 95% CI) and p values presented by country. [file 12916_2021_1985_MOESM5_ESM.docx]

**Table S4. Univariable linear regression of 25(OH)D concentrations by study variables in each country**

|  | **Kenya** | | **Uganda** | | | | **Burkina Faso** | | **The Gambia** | | **South Africa** | |
| --- | --- | --- | --- | --- | --- | --- | --- | --- | --- | --- | --- | --- |
|  | **Beta (95% CI)** | **P** | **Beta (95% CI)** | | **P** | | **Beta (95% CI)** | **P** | **Beta (95% CI)** | **P** | **Beta (95% CI)** | **P** |
| **Country** | Ref. | - | -0.04 (-0.07, -0.02) | | 0.0002 | | -0.06 (-0.10, -0.02) | 0.003 | -0.15 (-0.18, -0.12) | <0.0001 | 0.12 (-0.14, -0.09) | <0.0001 |
| **Age in months** | -0.09 (-0.10, -0.08) | <0.0001 | -0.07 (-0.09, -0.05) | | <0.0001 | | -0.20 (-0.26, -0.14) | <0.0001 | 0.002 (-0.02, 0.020) | 0.83 | -0.22 (-0.45, 0.01) | 0.06 |
| **Sex: females** | -0.01 (-0.05, 0.02) | 0.42 | -0.003 (-0.03, 0.03) | | 0.87 | | 0.02 (-0.04, 0.08) | 0.42 | -0.04 (-0.09, -0.003) | 0.036 | -0.02 (-0.07, 0.02) | 0.29 |
| **Year seasons^#^** |  |  |  | |  | |  |  |  |  |  |  |
| Summer/short rains/dry | Ref. | - | Ref. | | - | | Ref. | - | - | - | Ref. | - |
| Autumn/dry | 0.06 (0.02, 0.10) | 0.01 | 0.02 (-0.03, 0.06) | | 0.43 | | 0.08 (-0.004, 0.16) | 0.062 | - | - | 0.08 (0.005, 0.15) | 0.036 |
| Winter/long rains | 0.05 (-0.03, 0.13) | 0.22 | -0.08 (-0.12, -0.03) | | 0.001 | | -0.03 (-0.11, 0.05) | 0.42 | Ref. | - | -0.18 (-0.25, -0.12) | <0.0001 |
| Spring/dry | 0.06 (-0.01, 0.14) | 0.10 | -0.02 (-0.06, 0.03) | | 0.48 | | 0.02 (-0.23, 0.27) | 0.90 | 0.01 (-0.05, 0.06) | 0.86 | -0.12 (-0.18, -0.06) | <0.0001 |
| **Nutritional status** |  |  |  | |  | |  |  |  |  |  |  |
| Stunting^†^ | -0.0002 (-0.08, 0.08) | 0.997 | 0.0003 (-0.04, 0.04) | | 0.99 | | 0.01 (-0.06, 0.07) | 0.85 | -0.03 (-0.08, 0.01) | 0.17 | n/a | n/a |
| Underweight**^‡^** | -0.05 (-0.13, 0.02) | 0.16 | 0.002 (-0.06, 0.06) | | 0.94 | | -0.01 (-0.09, 0.070) | 0.83 | 0 .02 (-0.04, 0.07) | 0.50 | n/a | n/a |
| Wasting**^§^** | -0.04 (-0.17, 0.09) | 0.51 | 0.05 (-0.03, 0.13) | | 0.21 | | 0.01 (-0.11, 0.13) | 0.88 | 0.12 (0.04, 0.20) | 0.003 | n/a | n/a |
| **Inflammation**^¶^ | 0.04 (-0.0002, 0.08) | 0.051 | 0.06 (0.03, 0.10) | | 0.001 | | 0.03 (-0.03, 0.10) | 0.32 | 0.15 (0.09, 0.21) | <0.0001 | 0.12 (0.06, 0.18) | <0.0001 |
| **Malaria**^†^ | -0.16 (-0.21, -0.12) | <0.0001 | 0.04 (-0.03, 0.10) | | 0.25 | | -0.05 (-0.13, 0.03) | 0.20 | -0.01 (-0.08, 0.06) | 0.75 | n/a | n/a |
| **DBP Haplotype*** |  |  |  | |  | |  |  |  |  |  |  |
| Gc1f/f | Ref. | - | Ref. | - | | Ref. | | - | Ref. | - | Ref. | - |
| Gc1f/s | 0.03 (-0.03, 0.10) | 0.34 | 0.04 (-0.01, 0.09) | | 0.098 | | 0.06 (-0.03, 0.14) | 0.18 | 0.01 (-0.05, 0.07) | 0.80 | 0.001 (-0.08, 0.08) | 0.98 |
| Gc1f/2 | -0.10 (-0.16, -0.04) | 0.001 | -0.08 (-0.12, -0.04) | | 0.0003 | | -0.08 (-0.20, 0.04) | 0.18 | -0.08 (-0.18, 0.01) | 0.076 | -0.05 (-0.13, 0.019) | 0.15 |
| Gc1s/s | -0.07 (-0.29, 0.16) | 0.57 | 0.04 (-0.11, 0.18) | | 0.63 | | -0.04 (-0.43, 0.34) | 0.82 | 0.09 (-0.08, 0.26) | 0.30 | -0.03(-0.37, 0.31) | 0.88 |
| Gc1s/2 | -0.20 (-0.34, -0.06) | 0.006 | -0.08 (-0.19, 0.03) | | 0.16 | | -0.07 (-0.29, 0.16) | 0.56 | 0.09 (-0.10, 0.28) | 0.37 | 0.12 (-0.16, 0.39) | 0.41 |
| Gc2/2 | -0.17 (-0.45, 0.12) | 0.26 | -0.20 (-0.38, -0.01) | | 0.04 | | -0.33 (-0.88, 0.21) | 0.23 | - | - | -0.08 (-0.42, 0.26) | 0.65 |
| **Gc variant** |  |  |  | |  | |  |  |  |  |  |  |
| Gc1f | Ref. | - | Ref. | | - | | Ref. | - | Ref. | - | Ref. | - |
| Gc1s | -0.01 (-0.06, 0.05) | 0.85 | 0.03 (-0.01, 0.07) | | 0.13 | | 0.04 (-0.03, 0.12) | 0.31 | 0.03 (-0.02, 0.08) | 0.27 | 0.01 (-0.06, 0.08) | 0.79 |
| Gc2 | -0.11 (-0.16, -0.06) | <0.0001 | -0.08 (-0.12, -0.05) | | <0.0001 | | -0.10 (-0.13, 0.002) | 0.057 | -0.05 (-0.13, 0.03) | 0.23 | -0.04 (-0.11, 0.02) | 0.20 |
| Correlation coefficients and p values were obtained from linear regression analyses. 25(OH)D levels were ln-transformed to make them normally distributed. **^#^**Seasons were based on 3 monthly intervals. In South Africa the seasons are summer, autumn, winter and spring, in Uganda and Kenya there are two rainy seasons and in Burkina Faso and The Gambia there is a single rainy season. ^†^Stunting was defined as height-for-age Z score <-2; **^‡^**underweight as weight-for-age Z score <-2; **^§^** wasting as weight-for-height Z score < -2; ^¶^ inflammation as CRP >5 mg/L or ACT >0.6 g/L (ACT, but not CRP was available for The Gambia) and ^f^malaria as presence of *P. falciparum* parasites on blood film. *DBP haplotype was based on the combination of rs7041 and rs4588 genotypes. Gc variant was classified according to the individual alleles into Gc1f, Gc1s and Gc2. Percentage is based on the successfully typed SNPs, some participants’ genotype data was not available or failed QC. | | | | | | | | | | | | |
