# Supplementary material for: Prevalence and predictors of vitamin D deficiency in young African children
Source: BMC Med. 2021 May 20;19:115. doi: 10.1186/s12916-021-01985-8 (PMC8136043; doi:10.1186/s12916-021-01985-8)
Supplement: Supplementary file 7 — Additional file 7: Table S6. Median 25(OH)D levels by vitamin D binding protein haplotype and Gc variant in each country. This is a table of DBP variant and haplotype frequencies and median 25(OH)D levels by country. [file 12916_2021_1985_MOESM7_ESM.docx]

**Table S6. Median 25(OH)D levels by vitamin D binding protein haplotype and Gc variant in each country**

| **Combination of genotypes** | | |  | **Kenya** | | | |  | | **Uganda** | | |  | **Burkina Faso** | | |  | **The Gambia** | | |  | **South Africa** | | |
| --- | --- | --- | --- | --- | --- | --- | --- | --- | --- | --- | --- | --- | --- | --- | --- | --- | --- | --- | --- | --- | --- | --- | --- | --- |
| **rs7041** | **rs4588** | **DBP Haplotype*** |  | **n/total (%)** | **Median 25(OH)D nmol/L (IQR)** | **P*** |  | | **n/total (%)** | | **Median 25(OH)D nmol/L (IQR)** | **P*** |  | **n/total (%)** | **Median 25(OH)D nmol/L (IQR)** | **P*** |  | **n/total (%)** | **Median 25(OH)D nmol/L (IQR)** | **P*** |  | **n/total (%)** | **Median 25(OH)D nmol/L (IQR)** | **P*** |
| TT | CC | **Gc1f/f** |  | 671/962 (69.8%) | 79.2  (66.0, 97.0) | 0.006 |  | | 842/1288  (65.4%) | | 79.6  (66.0, 95.4) | 0.00010.0001 |  | 240/329  (72.9%) | 78.0  (64.0, 91.2) | 0.29 |  | 305/444  (68.7%) | 71.7  (60.0, 83.7) | 0.31 |  | 631/828  (76.2%) | 76.6  (60.5, 91.9) | 0.80 |
| TG | CC | **Gc1f/s** |  | 103/962  (10.7%) | 80.4  (67.2, 104.0) |  |  | | 177/1288  (13.7%) | | 83.2  (69.2, 96.8) |  |  | 56/329  (17.0%) | 82.0  (71.7, 95.4) |  |  | 91/444  (20.5%) | 71.7  (58.0, 85.0) |  |  | 87/828  (10.5%) | 74.7  (58.0, 91.9) |  |
| TT | CA | **Gc1f/2** |  | 154/962  (16.0%) | 71.4  (58.8, 92.2) |  |  | | 219/1288  (17.0%) | | 72.6  (62.5, 86.3) |  |  | 24/329  (7.3%) | 74.1  (59.7, 83.8) |  |  | 32/444  (7.2%) | 66.3  (51.6, 77.4) |  |  | 96/828  (10.5%) | 74.4  (59.7, 88.6) |  |
| GG | CC | **Gc1s/s** |  | 8/962  (0.8%) | 64.6  (50.8, 112.0) |  |  | | 15/1288  (1.2%) | | 82.3  (69.3, 98.8) |  |  | 2/329  (0.6%) | 76.4  (56.9, 95.9) |  |  | 9/444  (2.0%) | 87.4  (67.1, 91.6) |  |  | 4/828  (0.5%) | 74.7  (46.9, 115.8) |  |
| TG | CA | **Gc1s/2** |  | 21/962  (2.2%) | 70.3  (58.2, 89.0) |  |  | | 26/1288  (2.0%) | | 71.7  (59.4, 86.5) |  |  | 6/329  (1.8%) | 77.9  (56.6, 87.4) |  |  | 7/444  (1.6%) | 76.8  (63.3, 84.7) |  |  | 6/828  (0.7%) | 74.0  (61.8, 103.5) |  |
| TT | AA | **Gc2/2** |  | 5/962  (0.5%) | 75.7  (64.0, 79.7) |  |  | | 9/1288  (0.7%) | | 56.7  (52.4, 74.2) |  |  | 1/329  (0.3%) | 55.4 |  |  | 0  (0%) | - |  |  | 4/828  (0.5%) | 64.8  (55.7, 82.35) |  |
|  |  | **Gc variant^†^** |  |  |  |  |  | |  | |  |  |  |  |  |  |  |  |  |  |  |  |  |  |
| T | C | **Gc1f** |  | 1599/1924 (83.1%) | 79.0  (65.0, 97.0) | 0.0009 |  | | 2080/2576 (80.75%) | | 79.0  (65.7, 95.1) | 0.0001 |  | 560/658 (85.1%) | 78.3  (64.4, 91.3) | 0.0001 |  | 733/888 (82.55%) | 71.5  (59.1, 83.3) | 0.0001 |  | 1445/1656 (87.26%) | 76.1  (60.4, 91.6) | 0.0001 |
| G | C | **Gc1s** |  | 140/1924 (7.28%) | 79.1  (62.6, 100.0) |  |  | | 233/2576 (9.05%) | | 81.3  (68.8, 96.7) |  |  | 66/658 (10.0%) | 81.1  (68.9, 95.0) |  |  | 116/888  (13.06%) | 73.0  (59.0, 87.5) |  |  | 101/1656 (6.1%) | 74.7  (60.8, 91.9) |  |
| T | A | **Gc2** |  | 185/1924 (9.62%) | 71.7  (58.6, 90.0) |  |  | | 263/2576 (10.2%) | | 72.0  (60.4, 86.3) |  |  | 32/658 (4.86%) | 74.1  (56.0, 83.8) |  |  | 39/888  (4.39%) | 66.6  (53.7, 80.2) |  |  | 110/1656 (6.6%) | 73.5  (60.1, 88.7) |  |
| DBP; vitamin D binding protein; IQR, inter-quartile range; n/a, not available; 25(OH)D, 25-hydroxyvitamin D. *DBP haplotype is based on the combination of rs7041 and rs4588 genotypes. **^†^**Gc variant was classified according to the individual alleles into Gc1f, Gc1s and Gc2. Percentage is based on the successfully typed SNPs, some participants’ genotype data was not available or failed QC. ^*^P values were obtained by performing a Kruskal-Wallis equality-of-populations rank test to determine difference in 25(OH)D levels between DBP haplotypes or Gc variants. | | | | | | | | | | | | | | | | | | | | | | | | |
