# Supplementary material for: Prevalence and predictors of vitamin D deficiency in young African children
Source: BMC Med. 2021 May 20;19:115. doi: 10.1186/s12916-021-01985-8 (PMC8136043; doi:10.1186/s12916-021-01985-8)
Supplement: Supplementary file 8 — Additional file 8: Table S7. Summary characteristics of studies that evaluated the vitamin D status of African children (in alphabetical order). This is a table describing characteristics of past studies that evaluated the vitamin D status of young children in Africa. [file 12916_2021_1985_MOESM8_ESM.docx]

**Table S7. Summary characteristics of studies that evaluated the vitamin D status of African children (in alphabetical order)**

| **Author year** | **Study site, country** | **Sample size** | **Age mean (range/SD)** | **25(OH)D levels in healthy children^*^** | **Findings** |  |
| --- | --- | --- | --- | --- | --- | --- |
| Adegoke 2017 [48] | Ile-Ife, Nigeria | 75 healthy controls, HbAA (95 children with sickle cell disease, HbSS)^a^ | 7.35 (2.47) years | 112.9 (20.4) nmol/L | SCD patients had lower 25(OH)D levels than healthy controls. Daily vitamin D supplementation increased serum anti-inflammatory cytokine, IL-11, and lowered pro-inflammatory cytokines (IL-2, IL-6, IL-8, IL-17 and IL-18) in children with SCD. |  |
| Adegoke 2018 [49] | Ile-Ife, Nigeria | 30 healthy controls, HbAA (28 children with sickle cell disease, HbSS) | Range 1–15 years | 105.3 (25.0) nmol/L | Patients with SCD (HbSS) had lower 25(OH)D levels than healthy controls (HbAA). |  |
| Albanna 2010 [50] | Zagazig, Egypt | 40 healthy controls (40 children with pneumonia) | 3 (range 2–5) years | 87.2 (18.4) nmol/L | Healthy controls had higher vitamin D status than children with pneumonia. |  |
| Amukele 2012 [6] | Malawi | 21 HIV-exposed (uninfected) infants | 0 (range 0–1) years | 78.6 (10.4) nmol/L | Infants had 25(OH)D levels equal to their mothers at 12 months of age. |  |
| Aydemir 2014 [7] | Gazi University, Egypt | 20 healthy controls (40 children with sepsis) | Range 1–16 years | 69.9 (30.0) nmol/L | 25(OH)D levels were positively correlated with white blood cell count, CRP, TNF and IL-6 in all children. |  |
| Cusick 2014 [15] | Kampala, Uganda | 20 healthy controls (40 children with severe malaria) | 3.8 (range 1.5–12) years | 63.1 (21.7) nmol/L | Children with severe malaria had lower 25(OH)D levels than healthy community controls. There was no difference in 25(OH)D levels between children with cerebral malaria and those with severe malaria anemia. |  |
| Graff 2004 [51] | Jos, Nigeria | 15 healthy controls (15 children with rickets) | 4 (range 2–8) years | 72.4 (11.5) nmol/L | Children with rickets had lower 25(OH)D levels than non-rachitic children |  |
| Hamdy 2018 [52] | Cairo, Egypt | 60 healthy controls (80 SCD patients) | Range 4.3–15.5 years | 56.7 (21.0) nmol/L | Lower levels of 25(OH)D were associated with severe complications of SCD such as haemolysis. |  |
| Jones 2018[4] | Nairobi, Kenya | 22 urban community children (21 children with rickets) | 13 (range 2–24) months | 70 (54–85) nmol/L | 71% of the children had levels below 30 nmol/L. Children with rickets had lower 25(OH)D levels than non-rachitic children. Wasting, but not stunting or underweight, was associated with lower levels of 25OHD. |  |
| Nabeta 2015 [53] | Kampala, Uganda | 41 non-malnourished (117 malnourished children) | 1.3 (range 0.5–2.0) years | 80.4 (27.2) nmol/L | Malnutrition (<-2 SD weight-for-height) was not associated with 25(OH)D levels. |  |
| Oginni 1996a [54] | Ile-Ife, Nigeria | 94 healthy controls (44 children with rickets) | 3 (range 1–5) years | 63 (2.6) nmol/L | Children with rickets had lower 25(OH)D levels than non-rachitic children. |  |
| Oginni 1996b [55] | Ile-Ife, Nigeria | 20 healthy controls (22 children with rickets) | 3 (range 1–5) years | 69 (22) nmol/L | Children with rickets had lower 25(OH)D levels than non-rachitic children. |  |
| Pfitzner 1998 [8] | Jos, Nigeria | 198 community children (20 had rickets) | 2.0 (range 0.5–3.0) years | 64.9 (24) nmol/L | There was no difference in 25(OH)D levels between children with or without rickets. |  |
| Sudfeld 2015 [10] | Dar es Salaam, Tanzania | 948 HIV-exposed (uninfected) infants (253 HIV positive infants) | Range 1–24 months | 45.2 (23.0) nmol/L | Improved vitamin D status was associated with increased incidence of clinical and confirmed malaria. Stunting, wasting and underweight were not associated with vitamin D status. There was no difference in 25(OH)D levels between HIV positive and HIV negative infants. |  |
| Sudfeld 2017 [9] | Dar es Salaam, Tanzania | 581 infants born to HIV negative mothers | 0.3 (range 0.1–0.5) years | 64.9 (21.7) nmol/L | Exclusively breastfed infants were at higher risk of VDD than those receiving formula feeds. VDD was not associated with stunting, underweight, wasting, sex, birth order, birth weight, maternal age, maternal education, or household wealth. |  |
| Thacher 2000 [56, 57] | Jos, Nigeria | 123 healthy controls (123 children with rickets) | 4 (range 2–6) years | 51.2 (15.5) nmol/L | Children with rickets had lower 25(OH)D levels than non-rachitic children. |  |
| Tindall 2020 [58] | Botswana | 24 healthy controls | 4.0 years | 119.6 (7) nmol/L | Height was positively correlated with vitamin D status. |  |
| Toko 2016 [43] | Chulaimbo, Kenya | 54 infants (63 mothers) | Infants, 0 years | 64.9 (26.4) nmol/L | 25(OH)D levels did not differ in mothers and their infants with malaria parasitemia and those who were not infected. |  |
| Walter 1997 [59] | Jos, Nigeria | 27 healthy controls (16 children with rickets) | 3 (range 1–7) years | 59.9 (18.7) nmol/L | Children with rickets had lower 25(OH)D levels than non-rachitic children. |  |
| ^*^Mean (SD) or median (IQR) 25(OH)D levels presented only for healthy children. Only studies that reported mean 25(OH)D values for pre-school children were included in the meta-analysis (Figure 3). VDD, vitamin D deficiency, SD, standard deviation; CRP, C-reactive protein; 25OHD, 25-hydroxyvitamin D; SCD, sickle cell disease; HbSS, homozygous hemoglobin S; HbAA, normal hemoglobin; TNF, tumour necrosis factor; BMI, body mass index. | | | | | | |
