# Supplementary material for: Prevalence and predictors of vitamin D deficiency in young African children
Source: BMC Med. 2021 May 20;19:115. doi: 10.1186/s12916-021-01985-8 (PMC8136043; doi:10.1186/s12916-021-01985-8)
Supplement: Supplementary file 9 — Additional file 9: Figure S1. The precision profile of the 25(OH)D assay. This is a scatter plot of coefficient of variation (%) of the 25(OH)D assay used in this study. [file 12916_2021_1985_MOESM9_ESM.docx]

**Figure S1. The precision profile of the 25(OH)D assay.** The precision was assessed by 12-hourly internal quality control (IQC) sample runs at three concentration levels with the use of four separate lots of Technopath IQC reagents that had different target values. The data are from a period of 20 weeks during which the study was undertaken, with a total of 1,383 IQC samples run over 20 separate assay calibrations.
