# Supplementary material for: Prevalence and predictors of vitamin D deficiency in young African children
Source: BMC Med. 2021 May 20;19:115. doi: 10.1186/s12916-021-01985-8 (PMC8136043; doi:10.1186/s12916-021-01985-8)
Supplement: Supplementary file 10 — Additional file 10: Figure S2. Boxplots of 25(OH)D concentrations by country (A), age categories (B), sex (C), season (D) stunting (E), underweight (F), wasting (G), inflammation (H), malaria (I), vitamin D binding protein (DBP) isotype (J) and Gc variant (K). This is a grid of boxplots of 25(OH)D concentrations for each study variable. [file 12916_2021_1985_MOESM10_ESM.docx]

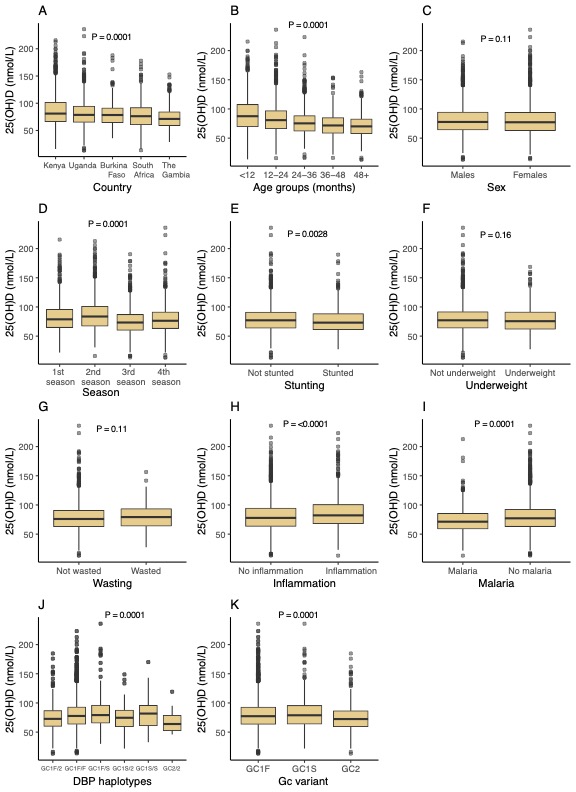


**Figure S2. Boxplots of 25(OH)D concentrations by country (A), age categories (B), sex (C), season (D) stunting (E), underweight (F), wasting (G), inflammation (H), malaria (I), vitamin D binding protein (DBP) isotype (J) and Gc variant (K).** Season was based on 3 monthly intervals. In South Africa the seasons are summer, autumn, winter and spring, in Uganda and Kenya there are two rainy seasons and in Burkina Faso and The Gambia there is a single rainy season. Stunting was defined as height-for-age Z score <-2; underweight as weight-for-age Z score <-2; wasting as weight-for-height Z score < -2; inflammation as CRP >5 mg/L or ACT >0.6 g/L (ACT, but not CRP was available for The Gambia) and malaria as presence of *P. falciparum* parasitaemia on blood film. P values were obtained by performing a Wilcoxon rank-sum test for variables with two categories and Kruskal-Wallis equality-of-populations rank test for variables with more than two categories.
