# Supplementary material for: Prevalence and predictors of vitamin D deficiency in young African children
Source: BMC Med. 2021 May 20;19:115. doi: 10.1186/s12916-021-01985-8 (PMC8136043; doi:10.1186/s12916-021-01985-8)
Supplement: Supplementary file 11 — Additional file 11: Figure S3. Boxplots of CRP levels overall and by country. These are boxplots of CRP levels. [file 12916_2021_1985_MOESM11_ESM.docx]

**Figure S3. Boxplots of CRP levels overall and by country.** The Gambia was not included as CRP measurements were unavailable.
