# Supplementary material for: Prevalence and predictors of vitamin D deficiency in young African children
Source: BMC Med. 2021 May 20;19:115. doi: 10.1186/s12916-021-01985-8 (PMC8136043; doi:10.1186/s12916-021-01985-8)
Supplement: Supplementary file 13 — Additional file 13: Figure S5. Meta-analysis of studies that estimated the prevalence of 25(OH)D levels < 75 nmol/L in healthy children aged 0–8 years in Africa. This is a meta-analysis plot of prevalence estimates of low vitamin D status (defined by 25(OH)D levels < 75 nmol/L) in young children included in the current and previous studies. [file 12916_2021_1985_MOESM13_ESM.docx]

**Figure S5. Meta-analysis of studies that estimated the prevalence of 25(OH)D levels <75 nmol/L in healthy children aged 0–8 years in Africa.** For eligible case-control studies, we included only prevalence estimates from healthy controls in the meta-analysis. Means or medians of age in months are presented. Studies that only measured 25(OH)D levels in cord blood or did not report estimates from young children separately were excluded from these analyses. Details of eligible studies are presented in Additional file 8: Table S7.
